# Analysis of Antimicrobial Resistance in Non-typhoidal *Salmonella* Collected From Pork Retail Outlets and Slaughterhouses in Vietnam Using Whole Genome Sequencing

**DOI:** 10.3389/fvets.2022.816279

**Published:** 2022-03-29

**Authors:** Niamh Holohan, Maximilian Wallat, Thi Hai Yen Luu, Eleanor Clark, Duong Thi Quy Truong, Sinh Dang Xuan, Hue Thi Kim Vu, Dung Van Truong, Hoang Tran Huy, Hung Nguyen-Viet, Fred Unger, Son Thi Thanh Dang, Richard A. Stabler

**Affiliations:** ^1^Department of Infection Biology, London School of Hygiene & Tropical Medicine, London, United Kingdom; ^2^Department of Bacteriology, National Institute of Veterinary Research (NIVR), Hanoi, Vietnam; ^3^International Livestock Research Institute, Regional Office for East and Southeast Asia, Hanoi, Vietnam; ^4^Department of Veterinary Hygiene, National Institute of Veterinary Research (NIVR), Hanoi, Vietnam; ^5^Department of Bacteriology, National Institute of Hygiene and Epidemiology, Hanoi, Vietnam

**Keywords:** non-typhoidal salmonella, antimicrobial resistance, colistin, *mcr*, pork, Vietnam, beta-lactamase, tetracycline

## Abstract

Non-typhoidal *salmonella* (TS) remains a significant health burden worldwide. In Vietnam, pork accounts for 70% of the total meat consumed, and contamination with *Salmonella* is high. High levels of antimicrobial resistance (AMR) have emerged among porcine NTS and of particular concern is the emergence of colistin resistance, a “last defense” antibioic against multi-drug resistant (MDR) Gram-negative pathogens. This study aimed to investigate the antibiotic susceptibility of 69 NTS isolates collected from the pork retail outlets and slaughterhouses in Vietnam during 2014 a nd 2018/19. Phenotypic testing and whole genome sequencing was used to assess the serotype and AMR gene profiles of the 69 NTS isolates. Seventeen different serotypes were identified, of which *S. enterica* subsp *enterica* serotype Typhimurium was the most common followed by *S*. ser. Rissen, *S*. ser. London, *S*. ser. Anatum, and *S*. ser. Derby. Phenotype AMR was common with 41 (59.4%) isolates deemed MDR. MDR strains were most common in slaughterhouses (83%) and supermarkets (75%) and lowest in traditional markets (38%) and convenience stores (40%). Colistin resistance was identified in 18 strains (15 resistant, three intermediate) with *mcr-1* identified in seven isolates (*S*. ser. Meleagridis, *S*. Rissen, *S*. Derby) and *mcr-3* in two isolates (*S*. Typhimurium). This includes the first *mcr* positive *S*. Meleagridis to our knowledge. Surprisingly, boutique stores had high levels (60%) of MDR isolates including 5/20 isolates with *mcr*-1. This study demonstrates that pork from modern retail stores classed as supermarkets or boutique (with pork claiming to be high quality, traceable, environmentally friendly marketed toward higher income consumers) still contained NTS with high levels of AMR.

## Introduction

Diarrhoeal disease remains a significant health burden worldwide causing approximately 45.5 million disability adjusted life years (DALYs) and an estimated 500,000 deaths in children under 5 years of age in 2019 (1). Non-typhoidal *salmonella* (NTS) is one of the key etiological agents of diarrhoeal disease, along with *Escherichia coli, Campylobacter*, and Norovirus (2), and results in an estimated 93.8 million cases of gastroenteritis and 155,000 associated deaths per annum (3).

*Salmonella* are Gram-negative bacteria of the family *Enterobacteriaceae*. Within two species, *S. bongori* and *S. enterica*, over 2500 different serotypes or serovars have been identified to date. *S. enterica* subsp. *enterica* contains 99% of serotypes responsible for human and animal infections (4). Over 95% of NTS infections are due to foodborne transmission (5), and the infection usually manifests as self-limiting gastroenteritis that does not require antimicrobial treatment. However, infection can also result in invasive disease, when the bacteria crosses the intestinal barrier into the bloodstream (6). The mortality rates of invasive disease is high and, thus, antimicrobial therapy is required (6). Recommended antimicrobial therapy regimens to treat invasive NTS infections are reviewed in (7).

Antimicrobial resistance (AMR) within NTS is now widespread, significantly contributing toward the public health burden [reviewed in (7)]. One contributing factor of emerging AMR has been antimicrobial use (AMU) for therapy, metaphylaxis, prophylaxis, and growth promotion in the animal production industry (8). Antimicrobials such as tetracycline, penicillins, and macrolides have been used as livestock growth promoters and prophylaxis since the 1950s (9). High proportions of NTS isolates from humans have shown resistance to antimicrobials that are linked to agricultural use, such as sulphonamides (32.8%), tetracyclines (30.2%), and ampicillin (27.5%) (10). In Europe by 2017, despite a ban on growth promotion, multi-drug resistance (MDR) among NTS isolates was high overall in clinical, fattening pigs and calves (28%) and was most common among *S. enterica* subsp. *enterica* serotype Typhimurium (11). Monitoring of *S*. Typhimurium from the food chain between 1996 and 2016 in China identified that 63% of over 11,000 isolates demonstrated resistance to at least four antibiotics including ampicillin, streptomycin, sulphonamides, and tetracycline (12). The percentage of multi-drug resistance in animals specifically was demonstrated to have increased between 2008 and 2016 (12). A systematic review of NTS in China identified an increasing prevalence of NTS in pork over time with high rates of tetracycline (0.68, CI 0.59–0.77), ampicillin (0.43, CI 0.34–0.53), streptomycin (0.42, CI 0.29–0.56), and sulfamethoxazole (0.42, CI 0.25–0.60) resistance (13). MDR has been associated with multiple serotypes such as Agona, Anatum, Dublin, Derby, Indiana, and Typhimurium (14–16).

Colistin, or polymyxin E, is a bactericidal antibiotic of the polymyxin family that targets the lipopolysaccharide (LPS) of the bacterial cell wall. It is primarily used as a last-resort drug against infections caused by MDR gram-negative bacteria (17). Resistance to colistin can occur as a result of mutations in chromosomal genes that alter molecular mechanisms associated with LPS [reviewed in (18)]. The most common modifications associated with resistance are commonly attributed to mutations in genes associated with the two component systems (TCS) *phoP/phoQ* and *pmrA/pmrB* (19) and genes directly involved in the biosynthesis of lipid A [*lpxA, lpxC*, and *lpxD* (20)]. Resistance can also occur through the acquisition of transposable genetic elements carrying mobilized colistin resistance (*mcr*) genes which modify lipid A (21). To date, at least ten *mcr* genes have been identified that are associated with a resistant phenotype [reviewed in (22)]. While *mcr* genes are typically found on plasmids, there have been reports of chromosomally encoded *mcr-1* and *mcr-3* in *Enterobacteriaceae* (23, 24). The most prevalent *mcr*-carrying species include *E. coli, S. enterica*, and *K. pneumonia* (25). Since discovery, *mcr* genes have been identified in at least 47 countries across six continents: Asia, Europe, North and South America, Africa, and Oceania (26). The earliest *mcr* positive bacterial strain, isolated from a human in Vietnam, was a *mcr-1* positive *Shigella sonnei*, isolated from a hospitalized child in Ho Chi Minh city in Vietnam in 2008 (27). In 2018, a study of *mcr-1* carrying isolates from 31 countries reported the largest number of positive isolates in China (46%), Vietnam (13%), and Germany (5%) (28).

Vietnam is a rapidly developing country with a population of 95.5 million. In recent years the country has seen a rapid economic transition and accelerated urbanization. However, Vietnam is receiving increasing interest due to the high burden of infectious disease and levels of AMR. Pork accounts for over 70% of total meat consumed in Vietnam and is estimated at nearly 24.7 kg/capita/year (29). As it is mainly produced by small farm holders and sold at traditional markets, the pig production sector provides a livelihood for 4.13 million farmers (29). NTS is an important cause of pediatric gastroenteritis in Vietnam and has been reported as the most prevalent pathogen in children hospitalized with diarrhea (30). This is unsurprising considering the high contamination rates on farms and on livestock products. Farm-level NTS prevalence of 64.7%, 94.3%, and 91.3% has been reported for chicken, duck, and pig, respectively (31). Furthermore, high AMR levels have been reported in NTS in retail meats, with the most prevalent resistance to tetracycline (58.5%), sulphonamides (58.1%), streptomycin (47.3%), ampicillin (39.8%), chloramphenicol (37.3%), and trimethoprim (34.0%) (32). Until recently, antimicrobial use for agriculture was widespread in Vietnam, and antimicrobials were a component of many animal feeds (33). In 2016, 55.4% and 42.2% of commercial pig and chicken feeds, respectively, contained at least one antimicrobial (34). Pig feeds were reported to contain antimicrobials considered of critical and high importance by the World Health Organization (WHO), such as chlortetracycline (23.9%), colistin (12.1%), amoxicillin (1.1%), and neomycin (0.9%) (34).

The presence of *Salmonella* in the pork production chain poses a serious public health risk. It has been suggested that the risk of NTS infection is increasing because of poorer hygiene and contamination control practices during the production process (35). To combat this issue, the Vietnamese government introduced a National Action Plan to monitor AMR in 2017 and a Law on Animal Husbandry in 2018 that aimed to eliminate antimicrobial use in animal feeds by 2020 (36). In 2016, the Vietnamese Ministry of Agriculture and Rural Development registered 69 shops as part of the “Green outlets – safe farm products program,” in an effort to address public concerns about food safety and provide products that meet safety requirements. These boutique stores sell pork claiming to be traceable, environmentally friendly, and of high quality, marketed toward high- and middle-income consumers. Since then, the number of boutique stores has been further increased across the country with some concentration across urban centers.

Two studies, PigRISK and SafePORK, have been funded by the Australian Center for International Agricultural Research to help improve food safety in Vietnam. The PigRISK project was conducted from 2012 to 2017 with the main objective to assess food safety risk and disease burden for pork consumers in smallholder pig value chains in Vietnam (37). In this study based in the Vietnam provinces of Hung Yen and Nghe An, pig products and environmental samples were collected from farms, slaughterhouses, and traditional markets. The SafePORK project is an ongoing project (2017–2022) which seeks to reduce the burden of food-borne illnesses in Vietnam highlighted by the PigRISK project (38, 39). The SafePORK project included determination of NTS prevalence in Vietnamese retail, including modern retail such as supermarkets, convenience stores, and boutique stores in Hanoi (38). Supermarkets were defined as large stores which sell foods and household goods; convenience stores are defined as small retail shops which sell everyday items including groceries, toiletries, and soft drinks; and boutique stores promote the so-called “safe agricultural products” including pork (40). SafePORK aims to contribute to the development of a One Health food safety programme in Vietnam and has highlighted the importance of cost-effective food safety practices along the pork production chain. It has suggested that greater regulation and surveillance around food safety procedures, as well as an increased budget for food safety interventions, is needed to improve food safety in Vietnam (41).

This study describes the use of whole genome sequencing (WGS) and bioinformatic analysis of 69 NTS isolates from the PigRISK (2014/15) and SafePORK (2018/19) studies. The isolates represent the pig slaughterhouses and pork retail (traditional markets, convenience stores, supermarkets, and boutique stores) and compares antibiotic susceptibility testing (AST) data across retail outlets. We performed phylogenetic analysis to correlate and confirm serotype predictions. The aim of the study was to investigate whether changes in meat retail from markets to modern specialist outlets have resulted in NTS serotypes with lower levels of AMR. We identified that despite the improved food security associated with modern retail, NTS from these locations had high levels of AMR including NTS containing resistance to a drug of last resort, colistin.

## Materials and Methods

### Study Design

The NTS strains in this study were kindly provided from the PigRISK and SafePORK collections. The PigRISK study identified NTS in 435/1275 samples (33.9%): 68/216 (31.5%) from farms, 182/545 (33.4%) slaughterhouses, and 185/514 (35.4%) traditional markets between April 2014 and February 2015. A total of 293/435 NTS had serotyping data which identified 30 serotypes. The most common PigRISK serotypes were *S*. Typhimurium (109, 37.2%), *S*. Derby (52, 17.7%), *S*. London (31, 10.6%), *S*. Rissen (15, 5.1%), *S*. Anatum (12, 4.1%), *S*. Weltevreden (9, 3.1%), *S*. Stanley (7, 2.4%), *S*. Enteritidis (5, 1.7%) and Unknown (15, 5.1%). Nineteen PigRISK isolates were selected to represent slaughterhouses (6 isolates) and traditional markets (13) and common serotypes: *S*. Typhimurium (9), *S*. Derby (1), *S*. London (1), *S*. Rissen (1), *S*. Weltevreden (1), *S*. Stanley (1), *S*. Enteritidis (3), and Unknown (2) (Supplementary Table 1).

SafePORK identified NTS from 404/705 (57.3%) samples including 167/328 (50.9%) from modern retail samples. SafePORK isolates were not serotyped prior to this study, therefore 50 isolates were selected that represented boutique shops (20 from 47 isolates, supermarkets (21/59), and convenience stores (9/61) (Supplementary Table 1). The boutique stores isolates included an in-depth study of Hai Ba Trung (10 isolates) district plus representatives from Ba Dinh 3, Dong Da 2, and Ha Dong 5. An in-depth study of convenience stores and supermarket isolates was from the Cau Giay district of Hanoi due to greatest incidence of NTS in these retail outlets.

### Isolation and Identification of Strains

*Salmonella* isolation and serotyping were carried out following ISO-6579, 2017 as described previously and stored at −30°C in cryovials containing brain heart infusion and 10% glycerol (42). Isolates were resurrected on selective IRIS *Salmonella* agar (Biokar, France) at 37°C for 24 h and sub-cultured onto XLD agar (Merck, Germany) for 24 h at 37°C. Confirmatory serotyping was performed following the Kauffman and White scheme using both polyvalent and monovalent O and H antiserum (Biorad, Germany).

### Phenotypic Antimicrobial Susceptibility Assay

Selected *Salmonella* strains were tested for antibiotic susceptibility following the Kirby–Bauer disc diffusion test and the recommendations of the Clinical and Laboratory Standards Institute (CLSI) (43). Antibiotics were used as follows: ampicillin (AMP, 10 μg), gentamycin (GEN, 10 μg), trimethoprim (TMP, 5 μg), tetracycline (TET, 30 μg), nalidixic acid (NAL, 30 μg), ciprofloxacin (CIP, 5 μg), ceftriaxone (CRO, 30 μg), cefotaxime (CTX, 30 μg), piperacillin (PRL, 30 μg), nitrofurantoin (NIT, 100 μg), and chloramphenicol (CHL, 30 μg) (Oxoid, Basingstoke, UK). The interpretation of inhibition zones was performed according to European Committee on Antimicrobial Susceptibility Testing (EUCAST, breakpoint table v11.0) (44) and Clinical & Laboratory Standards Institute (CLSI) (43). In addition, micro-broth dilution assay was performed to identify colistin (COL) resistance. A series of dilutions of colistin sulfate (Sigma Aldrich, St Louis, MO, USA), from 32 μg/mL to 0.25 μg/mL, was used following manufacturer instruction. *E. coli* ATCC 25922 was used as a quality control strain.

### Whole Genome Sequencing and Bioinformatic Analysis

Total genomic DNA was extracted using the Wizard genomic DNA extraction kit (Promega, USA) and quantified using the Qubit dsDNA BR assay kit (Invitrogen, USA). The Nextera XT library (2 x 301 bp) prep kit (Illumina, USA) and the Nextera DNA Flex library (2 x 151 bp) prep kit (Illumina, USA) were used to prepare the sequence libraries as per manufacturer's protocol. The samples were sequenced on a MiSeq System (Illumina, USA) as per the recommended protocol. Raw sequence data was quality controlled using Trimmomatic v0.38 (45) with the following specifications; Leading: 3, Trailing: 3, SlidingWindow: 4:20, and Minimum length: 36. Quality control (QC) checks were performed using FastQC v0.11.8 (46). Fastq reads were mapped against reference sequences using BWA MEM with default settings (47) and viewed in Artemis and ACT (48, 49). *De novo* sequence assemblies were performed using Spades v3.13 (50) with default settings, a coverage cut-off of 20, and k-mer lengths of 21, 33, 55, 77, 99, and 111. Draft genome multi-fasta files were evaluated using Quast assessment tool v5.0.2 (51). Three SafePORK isolates with assemblies which had a total base pair (bp) +/- 15% of 4.7Mbp were removed from analysis. Contigs were ordered against a *S*. Typhimurium str. LT2 (accession AE006468) using ABACAS v1.3.1 using -dmbc settings (52). The resulting assemblies were polished using Pilon v1.22 with default settings (53) and annotation using Prokka v1.13 in gram negative mode (54).

The assembled contigs were screened for AMR genes using ABRicate (55) v1.0.1 and CARD (56), and NCBI AMRFinderPlus (57) databases, and combined. Putative plasmid replicons were identified using the ABRicate with the PlasmidFinder database (58). Colistin sensitive genotypes were obtained from *S*. Typhimurium strain LT2 (*lpxA, lpxC, lpxD, phoP/phoQ*) (59) and *S*. Typhi CT18 (*pmrA*/*pmrB*) (60). MLST profiles were determined using the software package MLST v2.16.1 from the draft assemblies (61). SeqSero v1.0.1 (62) was used to predict serotype using both paired-end fastq and draft assembly genome data. The BSR-Based Allele Calling Algorithm (chewBBACA) (63) and predetermined *Salmonella* schema were used to generate cgMLST profiles and paralog removal using alleles present in 95% of reference and Vietnamese isolate genomes. Allele profile data was used to generate a MSTree in Grapetree using wgMLST and default settings (64). Heatmaps were generated using the Morpheus website (https://clue.io/morpheus) with hierarchical clustering using Euclidean distance using average linkage method.

## Results

### Whole Genome Sequencing

All 69 isolates were fully sequenced (Table 1, Supplementary Table 2). The average draft genome size was ~4.845 Mbp with an average GC content of 51.98% (Table 1), consistent with the *S*. Typhimurium chromosome (59). The average number of coding sequences (CDS) was 4,695 with an average of 11 rRNA and 82 tRNA per genome (Table 1).

**Table 1:** Summary statistics for genome assemblies and annotation.

**Parameter**	**Avg**	**SD**	**Min**	**Max**
Reads^*^	833,852	420,956	292,250	2,653,637
Genome size (Mb)	4.85	0.12	4.50	5.18
No. of contigs	48	18	11	88
GC (%)	51.98	0.20	51.56	52.25
N50 (bp)	4,07,760	1,72,894	1,59,925	9,66,226
CDS	4,695	289	4,182	5,502
rRNAs	10.9	4.1	4	18
tRNAs	82	3.4	78	97

*All assembly statistics are based on contigs of size >/= 500 bp*.

* *Reads after Trimmomatic quality control. Avg, mean of 69 assemblies; SD, standard deviation; Min, minimum value; Max, maximum value*.

### *In silico* Serotyping

SeqSero has been demonstrated previously to accurately predict *Salmonella* serotypes with congruence of 81 (65) and 84% (66) compared to phenotypic testing. When taking into account ambiguous antigenic formulas which are shared by more than one serotype, accuracy was 98% (66). SeqSero was tested against 117 curated high-quality reference genomes (67) and correctly predicted 115/117 (98.3%) isolates (Supplementary Table 3).

The majority of PigRISK isolates (17/19) had been previously serotyped using the traditional Kauffman and White method (68) and correlated with the SeqSero prediction (Supplementary Table 1). The unknown 2/19 isolates (PS121 & PS258) serotypes were predicted to be *S*. London and *S*. Weltevreden respectively (Supplementary Table 1).

**Figure 1:**
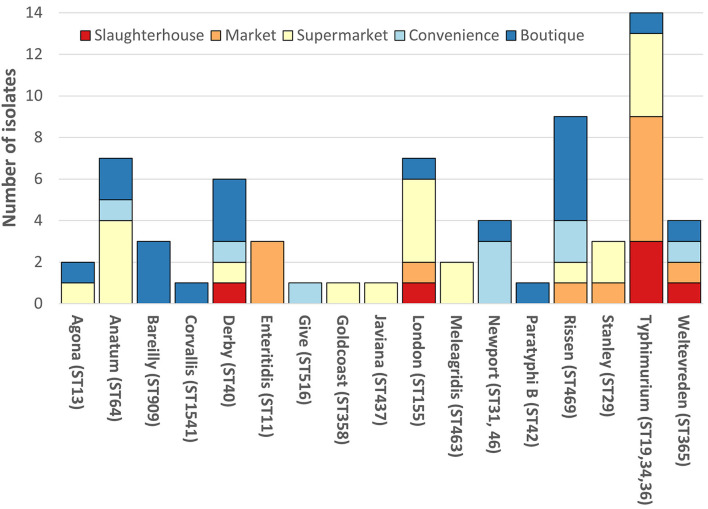
Breakdown of non-typhoidal salmonella (NTS) serotypes. Each vertical bar represents serotype with multi-locus sequence type(s) given in brackets. NTS serotypes by isolation location; red, slaughterhouse; orange, market; yellow, supermarket; light blue, convenience store; dark blue, boutique store. SafePORK serotype *in silico* predicted, PigRISK phenotypically serotyped and corroborated by *in silico* analysis. Corvallis, Corvallis or Chailey; Goldcoast, S. Goldcoast or *S*. Brikama; Javiana, II 9,12:l,z28:1,5 or *S*. Javiana, Typhimurium includes *S*. Typhimurium and potential monophasic variant of *S*. Typhimurium. ST34 includes SL406 a single SNP variant in *purE* of ST34.

The SafePORK study serotypes were unknown prior to strain selection (Supplementary Table 1). Convenience stores NTS *in silico* analysis identified 6 serotypes (from nine strains) including *S*. ser. Newport (3/9) and *S*. Rissen (2/9) (Figure 1, Supplementary Table 1). Supermarket NTS predicted 10 serotypes (from 21 strains) including *S*. London (4/21) and *S*. Typhimurium (4/21) and *S*. Anatum (4/21) (Figure 1, Supplementary Table 1). Boutique stores NTS predicted 11 serotypes from 20 strains including 5/20 *S*. Rissen, 3/20 *S*. Bareilly, and 3/20 *S*. Derby (Figure 1, Supplementary Table 1). Boutique stores strains serotypes were subsequently confirmed phenotypically (Supplementary Table 1). Seventeen serotypes were determined and/or predicted across 69 NTS. No serotype was identified in all five sampling area categories although *S*. anatum was only found in modern retail (supermarkets, convenience stores, and boutique stores) and *S*. Rissen in all retail store categories but not slaughterhouses (Figure 1).

Overall, *S*. Typhimurium was the predominant serotype, accounting for 14/69 (20.3%) of isolates (Figure 1). *S*. Typhimurium comprised of three multi-locus sequence types (ST): ST19 (6/13), ST36 (2/13), and ST34 (4/13 + 1/13 SLV/STNew) (Figure 1, Supplementary Table 1). *S*. Typhimurium SL406 had a novel single SNP variant of ST34 *purE* allele potentially generating a novel allele and ST. *S*. Newport was linked to two STs: ST46 and ST31. ST46 and ST31 only share 2 alleles: *purE*-15 and *thrA*-12. All other serotypes were associated with a single ST (Figure 1, Supplementary Table 1).

### Core Genome MLST

**Figure 2:**
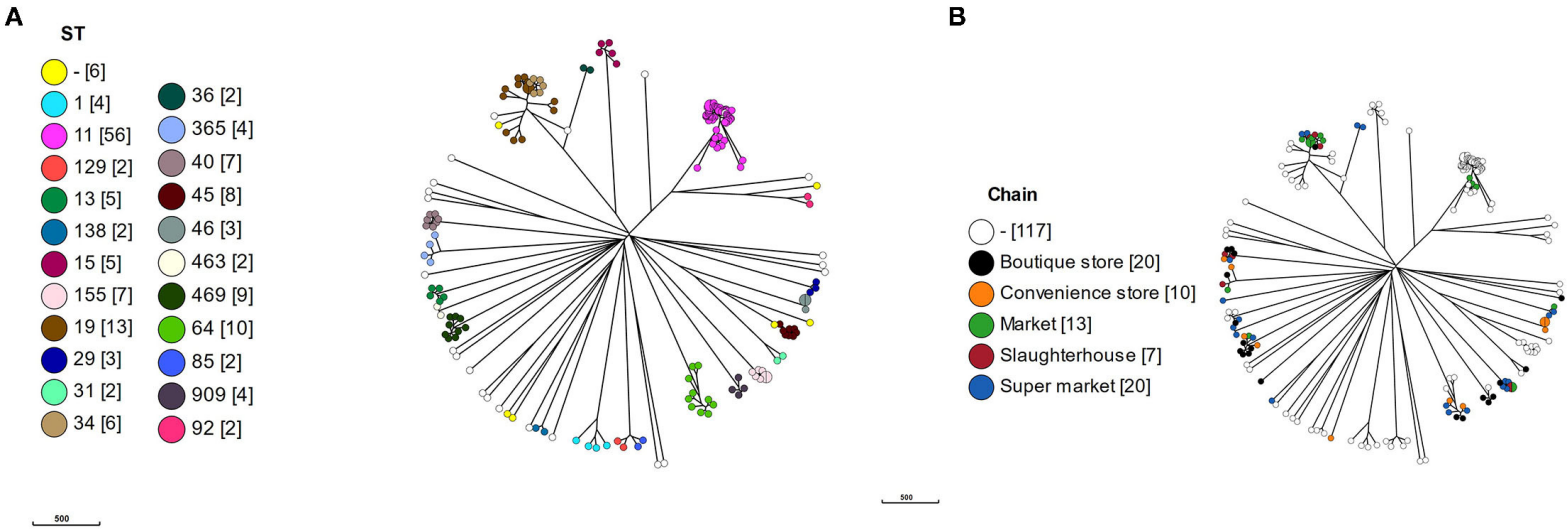
A minimal spanning tree based on core genome multi-locus sequencing type (cgMLST). A cgMLST schema was produced using a chewBBACA salmonella predetermined schema. The schema was used to call alleles in 117 reference and 69 Vietnamese isolates and a MSTree produced. **(A)** MSTree colored by MLST sequence type (ST) with number of isolates in square brackets, **(B)** MSTree colored by value chain location of isolates.

To put the Vietnamese isolates into context, cgMLST alleles were produced using a chewBBACA *Salmonella* predetermined schema for the 117 curated reference genomes plus the Vietnamese isolates. The cgMLST schema used 3190/8558 of the whole genome MLST (wgMLST) alleles based on 95% allele prevalence and removal of 182 paralogs. All isolates clustered according to ST (Figure 2A), which were proxy for serotypes, and the Vietnamese isolates were distributed throughout the tree (Figure 2B).

Within the *S*. Typhimurium isolates, the Vietnamese ST34 isolates formed a distinct cluster adjacent to the Vietnamese ST19 isolates. The Vietnamese *S*. Typhimurium ST19/ST34 isolates formed a separate cluster but adjacent to the reference ST19 isolates, suggesting a local variation in these isolates or reflecting difference between draft and completed genomes. The two Vietnamese ST36 isolates were deep rooted to the *S*. Typhimurium clade with the nearest reference isolate *S*. Typhimurium DT2 (ST128).

The *S*. Newport isolates formed three linked but distinct clusters defined by a single ST: ST31, ST46, and ST45. There was no reference ST46, however the three Vietnamese ST46 isolates were identical by cgMLST. The single Vietnamese ST31 was highly similar to reference CDC 2010K-2159, identified as a ST31. Eight reference ST45 *S*. Newport formed a cluster with *S*. Newport CVM N18486, a *purE* novel variant of ST45. *S*. Newport CVM 21554, a novel *aroC* variant of ST45, was the nearest neighbor to the ST45 cluster.

### Phenotypic Antimicrobial Resistance

**Figure 3:**
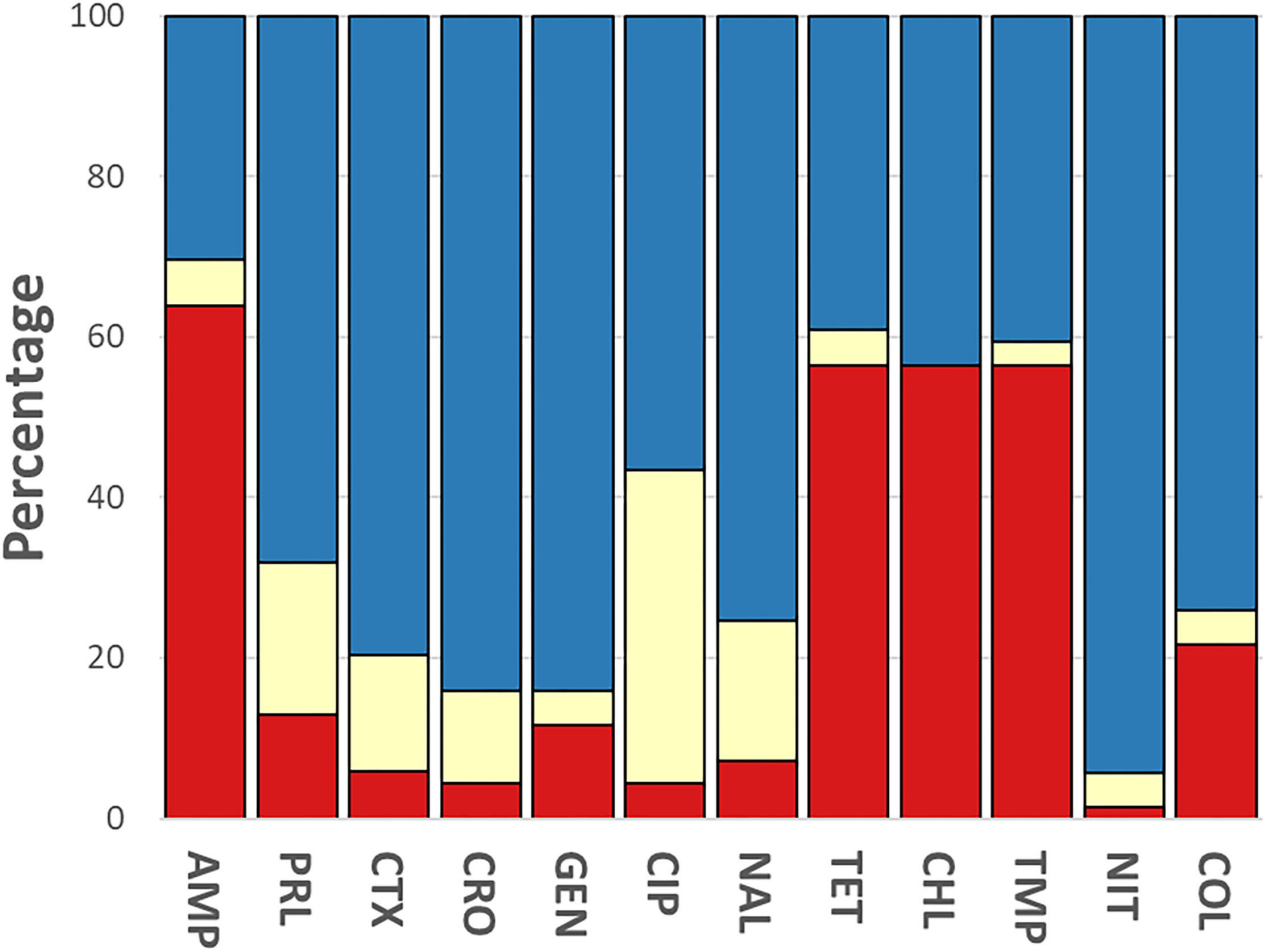
Breakdown of antimicrobial susceptibility testing for 69 isolates. Phenotypic resistance classified using EUCAST and CLSI guidelines. Red, resistant; yellow, intermediate; blue, susceptible. Antibiotics were used as follows: ampicillin (AMP, 10 μg), gentamycin (GEN, 10 μg), trimethoprim (TMP, 5 μg), tetracycline (TET, 30 μg), nalidixic acid (NAL, 30 μg), ciprofloxacin (CIP, 5 μg), ceftriaxone (CRO, 30 μg), cefotaxime (CTX, 30 μg), piperacillin (PRL, 30 μg), nitrofurantoin (NIT, 100 μg), chloramphenicol (CHL, 30 μg) (Oxoid, Basingstoke, UK).

**Figure 4:**
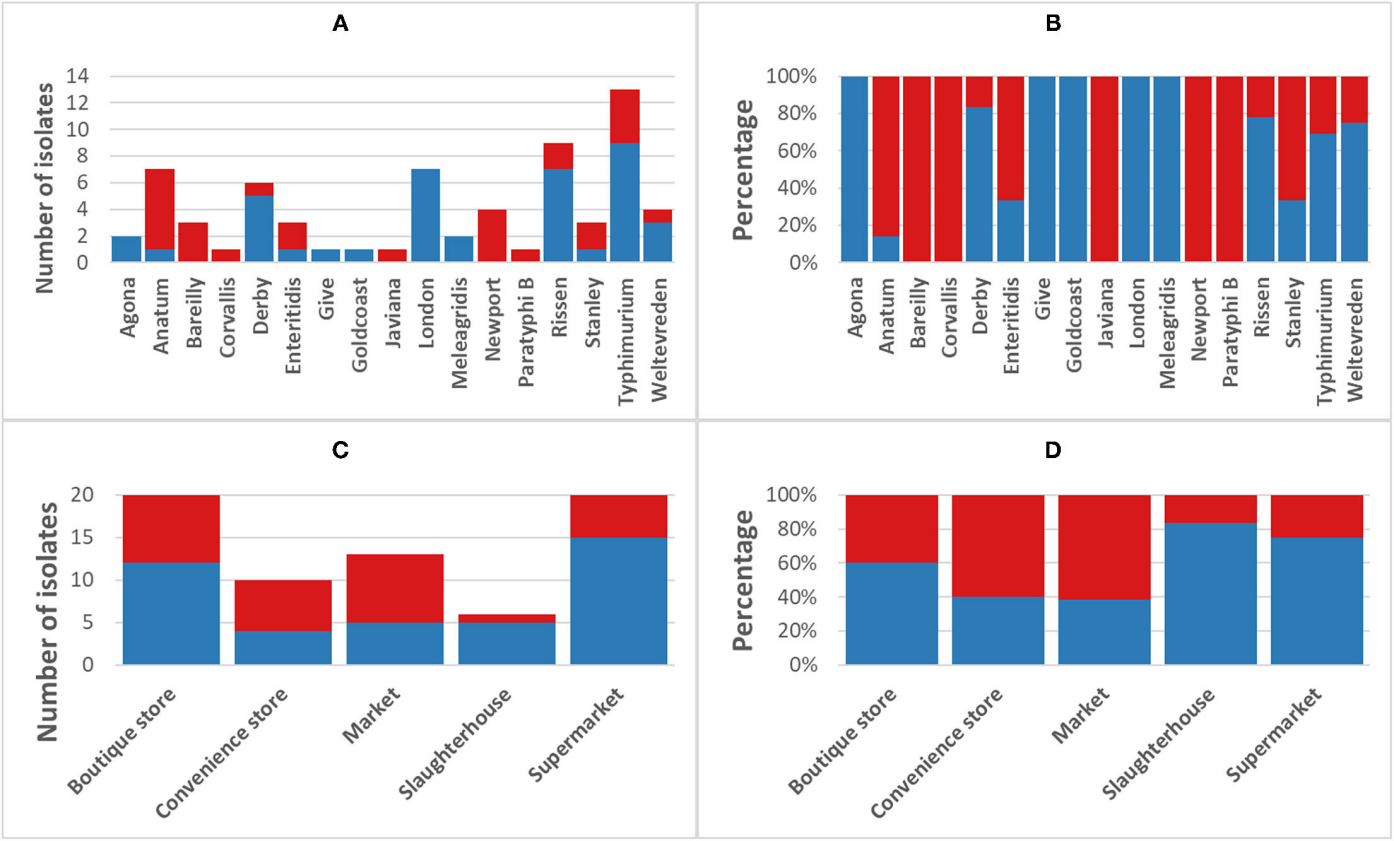
Incidence of multi-drug resistant non-typhoidal salmonella (NTS). Blue, NTS isolates classified as multi-drug resistant [MDR] (fully resistant to at least 1 antimicrobial from ≥3 different antimicrobial classes); Red, non-MDR isolates. **(A)** Breakdown of MDR/non-MDR isolates by serotype, **(B)** Breakdown of MDR/non-MDR isolates by serotype as a percentage, **(C)** Breakdown of MDR/non-MDR isolates source by isolation source, and **(D)** Breakdown of MDR/non-MDR isolates source by isolation source as a percentage.

The phenotypic disk diffusion/dilution results demonstrated that over half of the isolates were resistant or intermediately resistant to ampicillin (48, 69.6%), tetracycline (42, 60.9%), chloramphenicol (39, 56.5%), and trimethoprim (41, 59.4%) (Table 2, Figure 3, Supplementary Table 4). High prevalence of resistance was also seen against piperacillin (22, 31.8%), ciprofloxacin (30, 43.5%), and nalidixic acid (17, 24.6%) (Table 2, Supplementary Table 4). Resistant or intermediately resistant to third-generation cephalosporins, cefotaxime, and ceftriaxone was also high at 20.3 and 15.9%, respectively. Additionally, eighteen isolates (26.1%) were resistant to colistin (Table 2, Supplementary Table 4). MDR, defined as resistant to at least one antimicrobial from ≥3 different antimicrobial classes, was present in 41 (59.4%) isolates from twelve serotypes. All MDR isolates were resistant or intermediately resistant to ampicillin and/or piperacillin. All seven *S*. London (ST155) isolates were MDR (resistant to ampicillin/piperacillin, trimethoprim, and at least one other antimicrobial class) (Figure 4). The majority of *S*. Typhimurium isolates (9/13, 69.2%) were MDR (4/6 ST19, 4/6 ST34, 2/2 ST36, and SL406/ST34 variant) and were resistant or intermediately resistant to ampicillin/piperacillin and at least three other antimicrobial classes (Figure 4, Supplementary Table 4). Three *S*. Weltevreden (PS403, SP35, BD03-005), five *S*. Derby (SP164, SP9, HBT03-008, HBT03-014, HBT03-016), and both *S*. Meleagridis (SP90, SP130) isolates were MDR (Figure 4, Supplementary Table 4). Almost all isolates (65/69 94.2%) were susceptible to Nitrofurantoin, and a single *S*. Typhimurium slaughterhouse isolate was identified as resistant and two retail isolates with intermediate resistance (Table 2); PS234 (*S*. Typhimurium, ST19) was resistant and HBT03-001 (*S*. Rissen), SP136 (*S*. London), and SP164 (*S*. Derby) were intermediate resistant. SL406 (Typhimurium [ST34], slaughterhouse) was resistant to all four β-lactams, Aminoglycoside (Gentamicin), Tetracycline, Chloramphenicol, colistin, and intermediate resistant to quinolones (Nalidixic, Ciprofloxacin). A total of 5/69 (7.2%) isolates were fully susceptible to all 12 antimicrobials tested: SP1 [*S*. Anatum], SP46 & SP52 [*S*. Newport], SP97 [*S*. Stanley], and SP140 [*S*. Typhimurium] (Supplementary Table 4). Six (8.7%) isolates were only intermediately resistant to only one antimicrobial: PS281 (ciprofloxacin), PS411 (cefotaxime), SP22 (tetracycline), and SP30 (piperacillin), HBT03-002 & HBT03-005 (Ampicillin) (Supplementary Table 4). MDR strains were isolated from all four retail outlet categories and slaughterhouses (Figure 4). In slaughterhouses 5/6 (83%) were MDR, market 5/13 (38%), convenience stores 4/10 (40%), supermarkets 15/20 (75%), and boutique stores 12/20 (60%) (Figure 4).

**Table 2:** Summary of antimicrobial susceptibility testing for 69 isolates. Phenotypic resistance classified using EUCAST and CLSI guidelines.

		**Resistant**		**Intermediate**		**Susceptible**	
**Class**	**Antimicrobials**	**No. of isolates**	**%**	**No. of isolates**	**%**	**No. of isolates**	**%**
β-lactam	Ampicillin	44	63.8	4	5.8	21	30.4
	Piperacillin	9	13.0	13	18.8	47	68.1
	Cefotaxime	4	5.8	10	14.5	55	79.7
	Ceftriaxone	3	4.3	8	11.6	58	84.1
Aminoglycoside	Gentamycin	8	11.6	3	4.3	58	84.1
Quinolone	Ciprofloxacin	3	4.3	27	39.1	39	56.5
	Nalidixic acid	5	7.2	12	17.4	52	75.4
Tetracycline	Tetracycline	39	56.5	3	4.3	27	39.1
Phenol	Chloramphenicol	39	56.5	0	0	30	43.5
Folate pathway inhibitor	Trimethoprim	39	56.5	2	2.9	28	40.6
Nitrofuran	Nitrofurantoin	1	1.4	3	4.3	65	94.2
Cyclic peptide	Colistin	15	21.7	3	4.3	51	73.9

### Genotypic Antimicrobial Resistance

**Figure 5:**
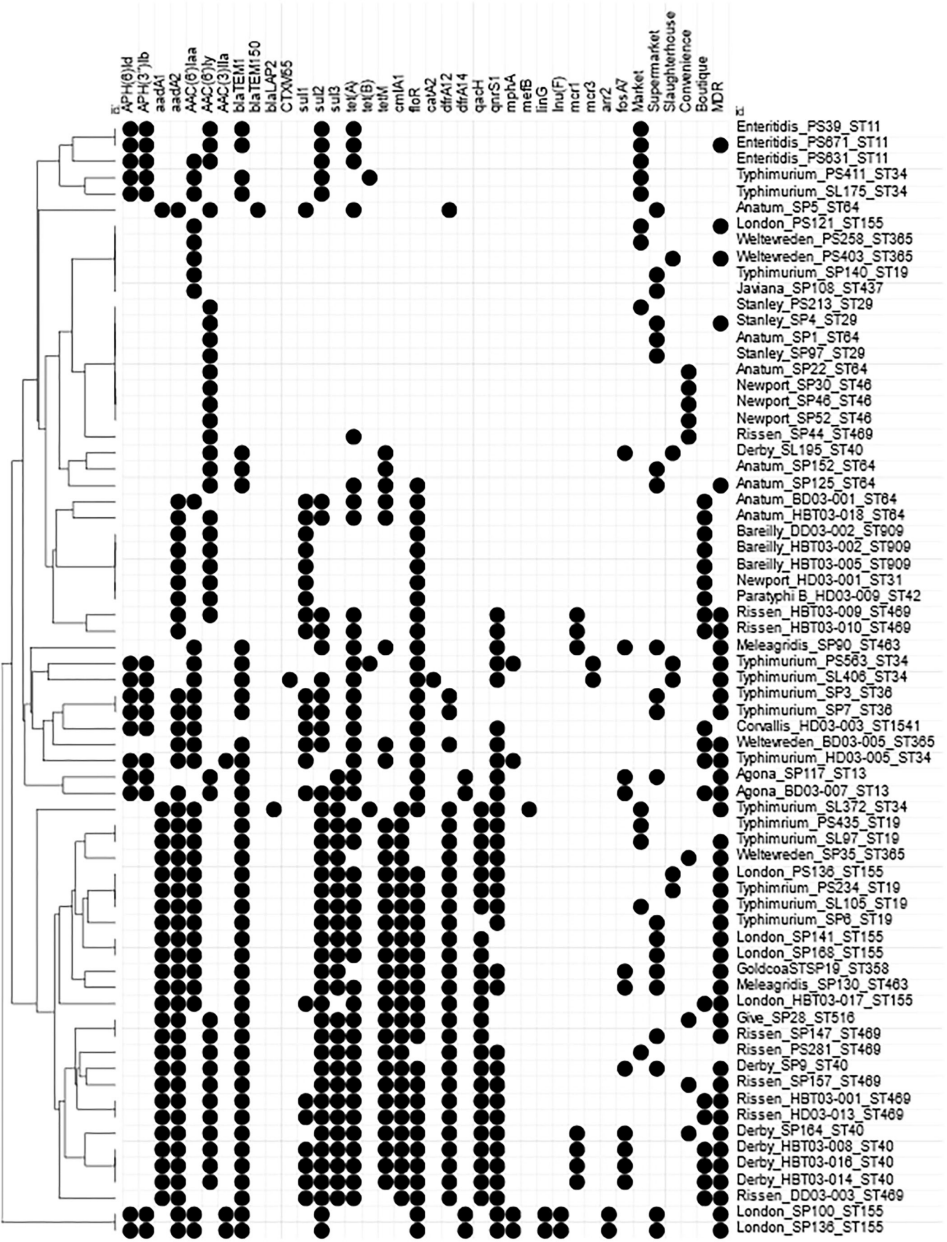
Heatmap of AMR genes within Vietnamese NTS isolates. Presence of Antimicrobial resistance genes within the draft genomes identified by Abricate using CARD and ResFinder databases. Circle indicates presence of match >90% identity with >90% coverage of reference gene. AMR genes present in all strains were removed; *golS, mdsABC, mktK*, and *sdiA*. MDR indicates an isolate defined as resistant to at least 1 antimicrobial from ≥3 different antimicrobial classes. Hierarchical clustered using Euclidian distance.

AMR genotypes were identified which are linked to resistance to 12 antibiotic classes, including β-lactams, tetracycline, and polymyxins. Genes associated with the multidrug efflux pumps MdsABC (*mdsA, mdsB, mdsC*, and *golS*) and MdtK (*mdtK*) were identified in 100% and 98.5% of isolates, respectively. The most common β-lactamase was *bla*_TEM−1_ identified in 43/69 isolates. TEM-1 is a broad-spectrum β-lactamase found in many Gram-negative bacteria which confers resistance to penicillins and first-generation cephalosporins. A total of 33/44 isolates that were resistant and 4/4 intermediate resistant to ampicillin possessed *bla*_TEM−1_; however, 6/21 ampicillin sensitive also contained a *bla*_TEM−1_. Additionally, piperacillin resistance (8/43) or intermediate resistance (9/43) was linked to TEM-1 positive strains. *bla*_TEM−150_ is a β-lactamase found in *Enterobacter* spp., *E. coli*, and *Klebsiella pneumoniae* as well as *Salmonella* and was identified in a single strain: SP5 (*S*. Anatum, supermarket), which was only ampicillin resistant. *bla*_LAP−2_ is an Ambler Class A β-lactamase gene conferring resistance to quinolones and was identified in SL372 (*S*. Typhimurium, market) which also was *bla*_TEM−1_ positive and demonstrated resistance to ampicillin, pipercillin, ceftriaxone, intermediate to cefotaxime, as well as the only strain nalidixic acid and ciprofloxacin resistant (Table 3, Figure 5, Supplementary Table 4). SL406 (*S*. Typhimurium, slaughterhouse) possessed *bla*_CTX−M−55_ as well as *bla*_TEM−1_ and was resistant to ampicillin, piperacillin, cefotaxime, and ceftriaxone (Figure 5, Supplementary Table 4). Trimethoprim resistance genes, *dfrA12* and *dfrA14*, were indicative of trimethoprim resistance; 30/39 (76.9%) of resistant isolates carried at least one *dfrA* gene and only 3/28 (10.7%) sensitive strains carried one of these genes. Chloramphenicol resistance genes (*floR* and *cmlA1*) were moderately indicative of chloramphenicol resistance. A total of 36/39 (92.3%) resistant strains possessed at least one resistance gene; however, 11/30 (36.7%) sensitive strains also possessed at least one resistance gene, with 2/30 (6.7%) containing both genes. Similarly, *qnrS1* presence, a fluoroquinolone resistance gene, was moderately linked to ciprofloxacin resistance, 3/3 (100%) resistant strains, and 21/27 (77.8%) intermediately resistant strains possessing the *qnrS1* gene. A total of 6/27 (22.2%) intermediately resistant strains did not possess *qnrS1* and 8/39 (20.5%) sensitive strains did possess the gene. The presence of genes linked to tetracycline resistance (*tetA, tetB*, and/or *tetM*) was not indicative of tetracycline resistance with 8/39 (20.5%) resistant strains possessing no identified *tet* gene and 15/27 (55.5%) susceptible strains possessing at least one *tet* gene, with 8/27 (29.6%) strains containing both *tetA* and *tetM*.

**Table 3:** Summary of AMR genes found in 69 non-typhoidal salmonella isolates from slaughterhouses and food retail outlet stores.

**Aminoglycoside**	**No. of isolates (Sl,M,C,Su,B)**		**β-Lactams**	**No. of isolates (Sl,M,C,Su,B)**		**Sulphonamides**	**No. of isolates (Sl,M,C,Su,B)**	
*aph6-ld*	15/69	(33, 38, 0, 25, 15%)	*TEM-1*	43/69	(**83**, 69, 50, 70, 50%)	*sul1*	23/69	(0, 0, 0, 15, **100%**)
*aph(3")-lb*	15/69	(33, 38, 0, 25, 15%)	*TEM-150*	1/69	(0, 0, 0, 5, 0%)	*sul2*	43/69	(50, 77, 40, 60, 70%)
*aadA1*	26/69	(33, 38, 40, 40, 35%)	*LAP-2*	1/69	(0, 8, 0, 0, 0%)	*sul3*	26/69	(33, 38, 40, 40, 35%)
*aadA2*	43/69	(33, 38, 40, 60, **100%**)	*CTX-M-55*	1/69	(17, 0, 0, 0, 0%)			
*aac(6')-laa*	32/69	(**83**, 69, 10, 60, 25%)						
*aac(6')-ly*	36/69	(17, 38, **90**, 40, 65%)						
*aac3-lla*	3/69	(0, 0, 0, 10, 5%)						
**Tetracycline**			**Chloramphenicol**			**Trimethoprim**		
*tet(A)*	42/69	(67, 54, 40, 60, 75%)	*cmlA1*	25/69	(33, 38, 40, 35, 35%)	*dfrA12*	29/69	(33, 38, 40, 50, 40%)
*tet(B)*	3/69	(17, 15, 0, 0, 0%)	*floR*	43/69	(67, 15, 30, 70, **100%**)	*dfrA14*	4/69	(0, 0, 0, 15, 5%)
*tet(M)*	31/69	(50, 31, 50, 45, 50%)	*catA2*	1/69	(17, 0, 0, 0, 0%)			
**Fluoroquinolones**			**Macrolides**			**Lincosamides**		
*qnrS1*	32/69	(67, 38, 30, 40, 60%)	*mphA*	4/69	(17, 0, 0, 10, 5%)	*linG*	2/69	(0, 0, 0, 10, 0%)
*qacH*	24/69	(33, 38, 40, 30, 35%)	*mefB*	1/69	(0, 8, 0, 0, 0%)	*Inu(F)*	2/69	(0, 0, 0, 10, 0%)
**Colistin**			**Rifampicin**			**Fosfomycin**		
*mcr-1*	7/69	(0, 0, 10, 5, 25%)	*arr-2*	2/69	(0, 0, 0, 10, 0%)	*fosA7*	11/69	(17, 0, 10, 25, 20%)
*mcr-3*	2/69	(33, 0, 0, 0, 0%)						

*Percentage of gene positive isolates given per sector (≥80% in bold); Sl, slaughterhouse (6 isolates); M, market (13); C, Convenience store (10); Su, Supermarket (20); B, Boutique store (20)*.

Mobile colistin resistance (*mcr*) genes were detected in nine isolates: *mcr-1* was present in *S*. Meleagridis (SP90), *S*. Rissen (HBT03-009, HBT03-010), and *S*. Derby (SP164, HBT03-008, HBT03-014, HBT03-016) (100% identity with *E. coli* SHP45 pHNSHP45 *mcr*-1 gene, accession NG_050417.1), *mcr-3* was present in PS563 (*S*. Typhimurium, ST34) and SL406 (*S*. Typhimurium, ST34 variant) (100% identity with *E. coli* WJ1 pWJ1 *mcr-3* gene, accession NG_055505.1). All nine of these isolates were colistin resistant (MIC 4 μg/ml except a single MIC 8 μg/ml for HBT03-010/*S*. Rissen/Boutique store). All nine of these isolates were determined MDR by AST, with SP90 showing resistance to four antimicrobial classes and SP164, PS563, and SL406 showing resistance to six antimicrobial classes.

MDR phenotype was associated with identification of multiple genes linked to AMR (Figure 5). A total of 33/69 strains with 10 or more (mean 12, range 10–16) AMR genes were MDR but two strains (*S*. Rissen, PS281 & *S*. Typhimurium, PS435) with 12 AMR genotypes were only intermediate resistant to Ciprofloxacin and intermediate resistant to ampicillin, cefotaxime, and ciprofloxacin, respectively. A total of 8/69 strains were MDR but had less than 10 (range 1–9) AMR genes.

### Identification of *mcr* Carrying Plasmids

The genomes of these isolates were screened for plasmid markers to predict potential *mcr* carrier plasmids. Incompatibility group (Inc) HI1A, IncHI1B, IncX4, IncFIA, and IncQ1 replicons were detected in SP90 and IncX4 replicons were detected in SP164. Furthermore, all *mcr-1* positive isolates carried IncX4 replicons (100% identity with the *E. coli* strain UMNF18 plasmid pUMNF18_32, accession CP002895) on the same contig as *mcr-1*. Assemblies and reads from these isolates mapped to an entire IncX4 reference plasmid pIMBC (MF449287), which contained *mcr-1* (Supplementary Figure 1). No plasmid replicons were detected in the *mcr-3* positive SL406 and, while IncA/C2 and IncQ1 replicons were detected in PS563, neither replicon was located on the same draft genome contig as *mcr-3*.

## Discussion

Food safety is of global concern and the common serotypes identified in this study have been linked to human illness in Vietnam (69, 70). In this study we used a collection of NTS from two studies of the pork value chain to understand the serotypes that are found on pork meat presented to customers, especially in context with increasing customer awareness and desire for safe food products. PigRISK and SafePORK both demonstrated that NTS are prevalent across all the retail outlets tested as well as at slaughterhouses.

WGS is now well-established as an essential tool for understanding bacterial pathogenesis, antimicrobial resistance, and epidemiology. This study, along with other studies (65, 66), demonstrates WGS can be used to reliably determine *Salmonella* serotypes either through direct *in silico* mimicking of the laborious laboratory method (SeqSero) or through a highly concordant proxy (MLST). Indeed, WGS derived core genome MLST (cgMLST) with hierarchical clustering has been proposed to replace phenotypic testing by defining major antigenic clusters as a ST-serovar linked to the Kauffmann and White scheme name (71). At present, cost and availability of both technology and reagents limit implementation of WGS in resource poor areas; however, this is rapidly changing.

Correct prediction of AMR phenotypes from WGS remains more elusive (72). Comparison of the AMR genotypes with AST data showed many inconsistencies with some isolates demonstrating resistance but without obvious genotypes and sensitive isolates having identifiable AMR genes within their genomes. Similar results of incoherence between AMR phenotype and genotype in *Salmonella* have been reported in China (73, 74). In this study, for example, the presence of TEM-1 was strongly linked to ampicillin resistance, although some *bla*_TEM−1_ positive strains were still sensitive. Hyperproduction of TEM-1 has been linked to piperacillin resistance (75), and in this study some TEM-1 positive strains also demonstrated resistance; however, prediction of expression levels by WGS is not easily determined. The difficulty in predicting AMR was in part due to complexity of interactions of multiple genotypes, annotation within AMR databases, focus on acquired genotypes over spontaneous mutations and difficulty in predicting expression and functionality of the proteins. For example, a study demonstrated that a *catA1* positive, chloramphenicol-sensitive *Salmonella* phenotype was attributed to a deleterious deletion in the promotor region (76). A further example of a sensitive porcine *E. coli* carrying an AMR plasmid with intact genes and promotors did not express the encoded phenotype even following inoculation into piglets, but the AMR phenotype was restored after the plasmid was introduced into a different strain (77). However, the carriage of unexpressed AMR genes in *Salmonella* has implications for public health as it suggests a reservoir of AMR genes, even in susceptible populations, that cannot be detected by phenotypic studies. Despite the challenges, improvements in prediction algorithms using WGS are become more common in diagnostic laboratories, for example, for prediction of resistance in *Mycobacteria tuberculosis*, and further development and may soon be suitable for other bacteria such as *Salmonella*.

*S*. Typhimurium was present in almost all animal-food sources, with monophasic variants (e.g., ST34) having a strong association with pork production (10). ST34 has been shown recently to be a pandemic clonal group associated with pigs and other wildlife and of global concern due to MDR isolates (78–81). *S*. Typhimurium was the most common serotype identified, with just over half of the sample (7/13, 54%) being ST34 variants. Two MDR *S*. Typhimurium ST34 isolates carried the *mcr-3*, a combination previously reported in Denmark, China, and Canada (82–84). Interestingly, cases in Denmark were linked to travel from Thailand and Vietnam (82) and cases in Canada were linked to travel from Thailand (84), supporting a suggestion that Southeast Asia is a potential reservoir for the global dissemination of *mcr-3* carrying *S*. Typhimurium ST34 (85). These slaughterhouses isolates were extremely drug resistant, having full or intermediate resistance to seven antimicrobial classes each. Concerningly, one isolate had also acquired a CTX-M-55 β-lactamase, complementing the TEM-1 already present, with an apparent increase in resistance to ceftriaxone. Treatment options for this strain would be limited with only sensitivity to nitrofurantoin of the antibiotics tested. Interestingly, two ST34 isolates were not resistant to any of the antibiotics.

*S*. Rissen has been identified as the most common serotype from pork samples from markets and supermarkets in Vietnam (86) and was the fourth most common isolate in the PigRISK study (slaughterhouses and markets). *S*. Rissen were isolated from all retail outlets in this study indicating that even with improved retail conditions and improvements in food production, this serotype remains a problem in the sector. *S*. Rissen has been linked to MDR phenotypes (87) and 7/9 *S*. Rissen in this study were MDR, including two isolates which possessed colistin resistance through *mcr*-1. An *mcr*-1 positive *S*. Rissen has been described in Vietnam previously (88) and *mcr*-1 was also found in 4/6 *S*. Derby isolates identified from modern retail, one from convenience store C02 (Cau Giay district) and three from boutique stores (B13, B14 & B15 within Hai Ba Trung district). The three isolates have near identical AMR phenotypes and identical genotypes suggesting a single strain and decoding retail outlets indicated these were three outlets for the same company. Additionally, the C02 isolate possessed the same genotypes as the boutique store isolates and highly similar AMR pattern despite the shop being a different company in a different district. This again indicates a possible common meat production network for these isolates, but further investigation of the network would confirm the WGS epidemiology.

The final *mcr*-1 positive strain (SP90) was in one of two *S*. Meleagridis NTS (Supermarket, Cau Giay district), and to our knowledge this is the first time this serotype has carried an *mcr* gene. Both *S*. Meleagridis NTS were from supermarkets and resistant to ampicillin, tetracycline, and chloramphenicol with SP90 having a Colistin MIC of 4 compared to 1 for SP130. Interestingly SP130 was also resistant to trimethoprim and intermediate to nalidixic acid.

The high levels of AMR in NTS from the pork retail outlets is concerning. Resistance (complete and intermediate) was observed to multiple antimicrobials considered critically important for human health by WHO (89), including ampicillin (69.6%), piperacillin (31.9%), cefotaxime (20.3%), ceftriaxone (15.9%), gentamycin (15.9%), ciprofloxacin (43.5%), and colistin (26.1%). In particular, resistance to ciprofloxacin and third-generation cephalosporins in NTS is of concern as these antimicrobials are used for the treatment of invasive salmonellosis in humans (90). Indeed, in 2008–09, the most commonly sold antibiotics in Vietnamese hospitals were oral second- and third- generation cephalosporins (91). This study reported 59.4% of isolates as MDR, similar to other reports of NTS from retail meats in Vietnam (86, 92), higher than the last EFSA report on MDR in *Salmonella* isolated from pigs (93) but lower than NTS isolates from pig slaughtering process in Hingzhou, China (85.9%) (73). MDR strains were isolates from all sample locations, with the highest proportions from slaughterhouses (5/6, 83.3%) and supermarkets (15/21, 71.4%). Boutique stores also showed a high prevalence of MDR strains (12/20, 60%), with 5 of these isolates carrying *mcr-1*, suggesting that while these stores promote “safe-agricultural products,” their pork produce may not be safer that those purchased from other retail outlets. Recently, the Vietnamese government, especially Ministry of Agriculture and Rural Development and Ministry of Health, has issued and updated the National Action Plan for combating drug resistance, which aims to promote prevention of drug resistance by improving the quality and effectiveness of the prevention and control measures, building surveillance capacity. One of the important components was to increase awareness on AMR among food and agriculture professionals, farmers, and the general public.

In Vietnam, pork is the most widely consumed meat representing more than 70% of all meat consumed. However, trust in food is low due to common food scares and continual high risk. Modern retail, especially boutique stores, arose to meet the concerns over food safety by promoting products as safe. In this study, while slaughterhouses and supermarkets had the highest levels of MDR strains, boutique stores were predicted to have lower levels of AMR due to the implied improved food production methods. In this study, however, NTS from boutique stores were often resistant to multiple classes of antibiotics, with 60% of isolates being classified as MDR. Interestingly, convenience stores and markets had lower rates of MDR. However, limitations to this study were the small number of isolates and sampled locations. The data presented here highlight the ongoing importance of AMR among NTS on retail meat despite the improvement in modern retail, with implications on human health irrespective of where the meat was purchased.

## Data Availability Statement

The datasets presented in this study can be found in online repositories. The names of the repository/repositories and accession number(s) can be found below: https://www.ebi.ac.uk/ena, PRJEB44046.

## Author Contributions

FU, ST, HN-V, and RS made substantial contributions to the conception and design of the work. NH, MW, TH, EC, DT, SX, HV, DV, HT, and RS aided the acquisition of the raw data. NH, MW, TH, EC, DT, SX, and RS analysis and interpretation of data. NH, MW, and RS wrote the manuscript. All authors have approved the submitted version.

## Funding

This work was partially supported by the CGIAR Research Program on Agriculture for Nutrition and Health (A4NH) and the Australian Center for International Agriculture Research (ACIAR, PigRISK LPS/2010/047 and SafePORK LPS/2016/143).

## Author Disclaimer

The opinions expressed here belong to the authors and do not necessarily reflect those of A4NH or CGIAR.

## Conflict of Interest

The authors declare that the research was conducted in the absence of any commercial or financial relationships that could be construed as a potential conflict of interest.

## Publisher's Note

All claims expressed in this article are solely those of the authors and do not necessarily represent those of their affiliated organizations, or those of the publisher, the editors and the reviewers. Any product that may be evaluated in this article, or claim that may be made by its manufacturer, is not guaranteed or endorsed by the publisher.

## Supplementary Material

Supplementary Figure 1 Comparison between *mcr*-1 positive non-typhoid Salmonella with reference *mcr*-1 plasmid plMBC (MF449287.2). CDS MF449287_004 (red) indicates location of *mcr*-1. **(A)** SP90 fastq reads mapped to pIMBC plasmid sequence; **(B)** ACT comparison of SP90 draft genome with pIMBC plasmid sequence; **(C)** SP164 fastq reads mapped to pIMBC plasmid sequence; **(D)** ACT comparison of SP164 draft genome with pIMBC plasmid sequence.

Supplementary Table 1 Non-typhoidal isolates from slaughterhouses and retail outlets. ID, Unique strain identifier; Study, research study through which the isolates were obtained; Market/Slaughterhouse ID, unique anonymised retail outlet identifier; Kauffman and White Serotype, phenotypic serotype; SeqSero_fastq, Predicted NTS serotype using SeqSero algorithm using fastq data; ST, Multilocus serotype (MLST) sequence type derived from draft genome assemblies; aroC/dnaN/hemD/hisD/purE/sucA/thrA, MLST schema allele identifViers.

Supplementary Table 2 Draft genome assembly data.

Supplementary Table 3 Reference genome data.

Supplementary Table 4 Antibiotic resistance. Phenotypic antimicrobial resistance to six classes of antibiotics were tested using CLSI guidelines. Draft genomes were analyzed using Abricate with a minimum coverage of 90% and minimum identity of 90% (displayed).

## Abbreviations

AMR: Antimicrobial resistance
AMU: Antimicrobial use
CLSI: Clinical & Laboratory Standards Institute
EUCAST: European Committee on Antimicrobial Susceptibility Testing
*mcr*: mobilized colistin resistance
MDR: Multi-drug resistance
NTS: Non-typhoidal *Salmonella*
LPS: Lipopolysaccharide
WHO: World Health Organization.

## References

[1] Hasso-Agopsowicz M Lopman BA Lanata CF Rogawski McQuade ET Kang G Prudden HJ. World health organization expert working group: recommendations for assessing morbidity associated with enteric pathogens. Vaccine. (2021) 39:7521–5. 10.1016/j.vaccine.2021.11.033 34838322

[2] Havelaar AH Kirk MD Torgerson PR Gibb HJ Hald T Lake RJ. World health organization global estimates and regional comparisons of the burden of foodborne disease in 2010. PLoS Med. (2015) 12:e1001923. 10.1371/journal.pmed.1001923 26633896 PMC4668832

[3] Majowicz SE Musto J Scallan E Angulo FJ Kirk M O'Brien SJ. The global burden of nontyphoidal Salmonella gastroenteritis. Clin Infect Dis. (2010) 50:882–9. 10.1086/650733 20158401

[4] Chan K Baker S Kim CC Detweiler CS Dougan G Falkow S. Genomic comparison of Salmonella enterica serovars and Salmonella bongori by use of an S. enterica serovar typhimurium DNA microarray. J Bacteriol. (2003) 185:553–63. 10.1128/JB.185.2.553-563.2003 12511502 PMC145314

[5] Mead PS Slutsker L Dietz V McCaig LF Bresee JS Shapiro C. Food-related illness and death in the United States. Emerg Infect Dis. (1999) 5:607–25. 10.3201/eid0505.990502 10511517 PMC2627714

[6] Hohmann EL. Nontyphoidal salmonellosis. Clin Infect Dis. (2001) 32:263–9. 10.1086/318457 11170916

[7] Tack B Vanaenrode J Verbakel JY Toelen J Jacobs J. Invasive non-typhoidal Salmonella infections in sub-Saharan Africa: a systematic review on antimicrobial resistance and treatment. BMC Med. (2020) 18:212. 10.1186/s12916-020-01652-4 32677939 PMC7367361

[8] Schwarz S Kehrenberg C Walsh TR. Use of antimicrobial agents in veterinary medicine and food animal production. Int J Antimicrob Agents. (2001) 17:431–7. 10.1016/S0924-8579(01)00297-7 11397611

[9] Angulo FJ Baker NL Olsen SJ Anderson A Barrett TJ. Antimicrobial use in agriculture: controlling the transfer of antimicrobial resistance to humans. Semin Pediatr Infect Dis. (2004) 15:78–85. 10.1053/j.spid.2004.01.010 15185190

[10] Authority EFS Prevention ECfD Control. The European Union summary report on trends and sources of zoonoses, zoonotic agents and food-borne outbreaks in 2017. EFSA J. (2018) 16:e05500. 10.2903/j.efsa.2018.5500 32625785 PMC7009540

[11] European Food Safety A European Centre for Disease P Control. The European Union summary report on antimicrobial resistance in zoonotic and indicator bacteria from humans, animals and food in 2017. EFSA J. (2019) 17:e05598. 10.2903/j.efsa.2019.5598 32626224 PMC7009238

[12] Wang X Biswas S Paudyal N Pan H Li X Fang W. antibiotic resistance in salmonella typhimurium isolates recovered from the food chain through national antimicrobial resistance monitoring system between 1996 and 2016. Front Microbiol. (2019) 10:985. 10.3389/fmicb.2019.00985 31134024 PMC6514237

[13] Shen W Chen H Geng J Wu RA Wang X Ding T. Prevalence, serovar distribution, and antibiotic resistance of Salmonella spp. isolated from pork in China: A systematic review and meta-analysis. Int J Food Microbiol. (2022) 361:109473. 10.1016/j.ijfoodmicro.2021.109473 34768041

[14] Chen S Zhao S White DG Schroeder CM Lu R Yang H. Characterization of multiple-antimicrobial-resistant salmonella serovars isolated from retail meats. Appl Environ Microbiol. (2004) 70:1–7. 10.1128/AEM.70.1.1-7.2004 14711619 PMC321239

[15] Lu Y Zhao H Sun J Liu Y Zhou X Beier RC. Characterization of multidrug-resistant Salmonella enterica serovars Indiana and Enteritidis from chickens in Eastern China. PLoS ONE. (2014) 9:e96050. 10.1371/journal.pone.0096050 24788434 PMC4008530

[16] Yang X Wu Q Zhang J Huang J Chen L Wu S. Prevalence, bacterial load, and antimicrobial resistance of salmonella serovars isolated from retail meat and meat products in China. Front Microbiol. (2019) 10:2121. 10.3389/fmicb.2019.02121 31608021 PMC6771270

[17] Loho T Dharmayanti A. Colistin: an antibiotic and its role in multiresistant Gram-negative infections. Acta Med Indones. (2015) 47:157–68. Available online at: http://actamedindones.org/index.php/ijim/article/view/52/48 26260559

[18] El-Sayed Ahmed MAE Zhong LL Shen C Yang Y Doi Y Tian GB. Colistin and its role in the Era of antibiotic resistance: an extended review (2000–2019). Emerg Microbes Infect. (2020) 9:868–85. 10.1080/22221751.2020.1754133 32284036 PMC7241451

[19] Stefaniuk EM Tyski S. Colistin resistance in enterobacterales strains - a current view. Pol J Microbiol. (2019) 68:417–27. 10.33073/pjm-2019-055 31880886 PMC7260631

[20] Moffatt JH Harper M Harrison P Hale JD Vinogradov E Seemann T. Colistin resistance in Acinetobacter baumannii is mediated by complete loss of lipopolysaccharide production. Antimicrob Agents Chemother. (2010) 54:4971–7. 10.1128/AAC.00834-10 20855724 PMC2981238

[21] Hu M Guo J Cheng Q Yang Z Chan EWC Chen S. Crystal Structure of Escherichia coli originated MCR-1, a phosphoethanolamine transferase for colistin resistance. Sci Rep. (2016) 6:38793. 10.1038/srep38793 27958270 PMC5153839

[22] Hussein NH Al-Kadmy IMS Taha BM Hussein JD. Mobilized colistin resistance (MCR) genes from 1 to 10: a comprehensive review. Mol Biol Rep. (2021) 48:2897–907. 10.1007/s11033-021-06307-y 33839987

[23] Zhou HW Zhang T Ma JH Fang Y Wang HY Huang ZX. Occurrence of Plasmid- and Chromosome-Carried mcr-1 in Waterborne Enterobacteriaceae in China. Antimicrob Agents Chemother. (2017) 61:17. 10.1128/AAC.00017-17 28559252 PMC5527621

[24] Ling Z Yin W Li H Zhang Q Wang X Wang Z. Chromosome-mediated mcr-3 variants in Aeromonas veronii from chicken meat. Antimicrob Agents Chemother. (2017) 61:17. 10.1128/AAC.01272-17 28848017 PMC5655048

[25] Elbediwi M Li Y Paudyal N Pan H Li X Xie S. Global burden of colistin-resistant bacteria: mobilized colistin resistance genes study (1980–2018). Microorganisms. (2019) 7:461. 10.3390/microorganisms7100461 31623244 PMC6843232

[26] Nang SC Li J Velkov T. The rise and spread of mcr plasmid-mediated polymyxin resistance. Crit Rev Microbiol. (2019) 45:131–61. 10.1080/1040841X.2018.1492902 31122100 PMC6625916

[27] Pham Thanh D Thanh Tuyen H Nguyen Thi Nguyen T Chung Chung The H Wick RR Thwaites GE . Inducible colistin resistance via a disrupted plasmid-borne mcr-1 gene in a 2008 Vietnamese Shigella sonnei isolate. J Antimicrob Chemother. (2016) 71:2314–7. 10.1093/jac/dkw173 27246235 PMC4954929

[28] Wang R van Dorp L Shaw LP Bradley P Wang Q Wang X. The global distribution and spread of the mobilized colistin resistance gene mcr-1. Nat Commun. (2018) 9:1179. 10.1038/s41467-018-03205-z 29563494 PMC5862964

[29] Nga NTD Lucy Lapar FU Pham Van Hung Duong Nam Ha Nguyen Thi Thu Huyen Tran Van Long. Household pork consumption behavior in Vietnam: Implications for pro-smallholder pig value chain upgrading. Conference on International Research on Food Security, Natural Resource Management and Rural Development. Berlin, German (2015).

[30] Thompson CN Phan MV Hoang NV Minh PV Vinh NT Thuy CT. A prospective multi-center observational study of children hospitalized with diarrhea in Ho Chi Minh City, Vietnam. Am J Trop Med Hyg. (2015) 92:1045–52. 10.4269/ajtmh.14-0655 25802437 PMC4426562

[31] Tu LT Hoang NV Cuong NV Campbell J Bryant JE Hoa NT. High levels of contamination and antimicrobial-resistant non-typhoidal Salmonella serovars on pig and poultry farms in the Mekong Delta of Vietnam. Epidemiol Infect. (2015) 143:3074–86. 10.1017/S0950268815000102 25778282 PMC4595858

[32] Thai TH Hirai T Lan NT Yamaguchi R. Antibiotic resistance profiles of Salmonella serovars isolated from retail pork and chicken meat in North Vietnam. Int J Food Microbiol. (2012) 156:147–51. 10.1016/j.ijfoodmicro.2012.03.016 22497836

[33] Pham-Duc P Cook MA Cong-Hong H Nguyen-Thuy H Padungtod P Nguyen-Thi H. Knowledge, attitudes and practices of livestock and aquaculture producers regarding antimicrobial use and resistance in Vietnam. PLoS ONE. (2019) 14:e0223115. 10.1371/journal.pone.0223115 31553776 PMC6760827

[34] Van Cuong N Nhung NT Nghia NH Mai Hoa NT Trung NV Thwaites G. Antimicrobial consumption in medicated feeds in vietnamese pig and poultry production. Ecohealth. (2016) 13:490–8. 10.1007/s10393-016-1130-z 27198232 PMC5063901

[35] Takeshi K Itoh S Hosono H Kono H Tin VT Vinh NQ. Detection of *Salmonella spp*. Isolates from specimens due to pork production Chains in Hue City, Vietnam *.J Vet Med Sci*. (2009) 71:485–7. 10.1292/jvms.71.485 19420853

[36] The Law on Livestock production (2018). Available online at: https://thukyluat.vn/vb/law-32-2018-qh14-prescribing-on-animal-husbandry-62e85.html#VanBanTA

[37] PigRisk: Reducing disease risks improving food safety in smallholder pig value chains in Vietnam. (2012). Available online at: https://aciar.gov.au/project/lps-2010-047

[38] SafePORK: Market Based Approaches to Improving the Safety of Pork in Vietnam. (2016). Available online at: https://aciar.gov.au/project/ls-2016-143

[39] Ngo HHT Nguyen-Thanh L Pham-Duc P Dang-Xuan S Le-Thi H Denis-Robichaud J. Microbial contamination and associated risk factors in retailed pork from key value chains in Northern Vietnam. Int J Food Microbiol. (2021) 346:109163. 10.1016/j.ijfoodmicro.2021.109163 33798966

[40] Overview of typical pork value chains in Vietnam 2019 [updated 11/11/21. Available online at: https://cgspace.cgiar.org/bitstream/handle/10568/102172/ResearchBrief_91.pdf

[41] Lam S Nguyen HTT Tuan HNH Nguyen LT Nguyen-Viet H Toribio JA. Unpacking the theory behind one health food safety programs: a vietnam case study. Front Vet Sci. (2021) 8:763410. 10.3389/fvets.2021.763410 34926640 PMC8672033

[42] Dang-Xuan S Nguyen-Viet H Pham-Duc P Unger F Tran-Thi N Grace D. Risk factors associated with *Salmonella spp*. prevalence along smallholder pig value chains in Vietnam. Int J Food Microbiol. (2019) 290:105–15. 10.1016/j.ijfoodmicro.2018.09.030 30317109

[43] CLSI M100| Performance Standards for Antimicrobial Susceptibility Testing. 28th Available online at: https://clsi.org/standards/products/microbiology/documents/m100/

[44] EUCAST: Clinical breakpoints and dosing of antibiotics. Available online art: https://eucast.org/clinical_breakpoints/

[45] Bolger AM Lohse M Usadel B. Trimmomatic: a flexible trimmer for Illumina sequence data. Bioinformatics. (2014) 30:2114–20. 10.1093/bioinformatics/btu170 24695404 PMC4103590

[46] Andrews S. FastQC: a quality control tool for high throughput sequence data {Online}. (2015). Available online at: https://qubeshub.org/resources/fastqc

[47] Li H Durbin R. Fast and accurate long-read alignment with Burrows-Wheeler transform. Bioinformatics. (2010) 26:589–95. 10.1093/bioinformatics/btp698 20080505 PMC2828108

[48] Carver T Harris SR Berriman M Parkhill J McQuillan JA. Artemis: an integrated platform for visualization and analysis of high-throughput sequence-based experimental data. Bioinformatics. (2012) 28:464–9. 10.1093/bioinformatics/btr703 22199388 PMC3278759

[49] Carver T Berriman M Tivey A Patel C Bohme U Barrell BG. Artemis and ACT: viewing, annotating and comparing sequences stored in a relational database. Bioinformatics. (2008) 24:2672–6. 10.1093/bioinformatics/btn529 18845581 PMC2606163

[50] Prjibelski A Antipov D Meleshko D Lapidus A Korobeynikov A. Using SPAdes De Novo Assembler. Curr Protoc Bioinformatics. (2020) 70:e102. 10.1002/cpbi.102 32559359

[51] Mikheenko A Prjibelski A Saveliev V Antipov D Gurevich A. Versatile genome assembly evaluation with QUAST-LG. Bioinformatics. (2018) 34:i142–i50. 10.1093/bioinformatics/bty266 29949969 PMC6022658

[52] Assefa S Keane TM Otto TD Newbold C Berriman M ABACAS. algorithm-based automatic contiguation of assembled sequences. Bioinformatics. (2009) 25:1968–9. 10.1093/bioinformatics/btp347 19497936 PMC2712343

[53] Walker BJ Abeel T Shea T Priest M Abouelliel A Sakthikumar S. Pilon: an integrated tool for comprehensive microbial variant detection and genome assembly improvement. PLoS ONE. (2014) 9:e112963. 10.1371/journal.pone.0112963 25409509 PMC4237348

[54] Seemann T. Prokka: rapid prokaryotic genome annotation. Bioinformatics. (2014) 30:2068–9. 10.1093/bioinformatics/btu153 24642063

[55] Seemann T. Abricate. Github, Available online at: https://github.com/tseemann/abricate.

[56] Jia B Raphenya AR Alcock B Waglechner N Guo P Tsang KK. CARD 2017: expansion and model-centric curation of the comprehensive antibiotic resistance database. Nucleic Acids Res. (2017) 45:D566–D73. 10.1093/nar/gkw1004 27789705 PMC5210516

[57] Feldgarden M Brover V Haft DH Prasad AB Slotta DJ Tolstoy I. Validating the AMRFinder tool and resistance gene database by using antimicrobial resistance genotype-phenotype correlations in a collection of isolates. Antimicrob Agents Chemother. (2019) 63:19. 10.1128/AAC.00483-19 31427293 PMC6811410

[58] Carattoli A Zankari E Garcia-Fernandez A Voldby Larsen M Lund O Villa L. In silico detection and typing of plasmids using PlasmidFinder and plasmid multilocus sequence typing. Antimicrob Agents Chemother. (2014) 58:3895–903. 10.1128/AAC.02412-14 24777092 PMC4068535

[59] McClelland M Sanderson KE Spieth J Clifton SW Latreille P Courtney L. Complete genome sequence of Salmonella enterica serovar Typhimurium LT2. Nature. (2001) 413:852–6. 10.1038/35101614 11677609

[60] Parkhill J Dougan G James KD Thomson NR Pickard D Wain J. Complete genome sequence of a multiple drug resistant Salmonella enterica serovar Typhi CT18. Nature. (2001) 413:848–52. 10.1038/35101607 11677608

[61] Seemann T. MLST. Github, Available online at: https://github.com/tseemann/mlst

[62] Zhang S Yin Y Jones MB Zhang Z Deatherage Kaiser BL Dinsmore BA. Salmonella serotype determination utilizing high-throughput genome sequencing data. J Clin Microbiol. (2015) 53:1685–92. 10.1128/JCM.00323-15 25762776 PMC4400759

[63] Silva M Machado MP Silva DN Rossi M Moran-Gilad J Santos S. chewBBACA: a complete suite for gene-by-gene schema creation and strain identification. Microb Genom. (2018) 4:166. 10.1099/mgen.0.000166 29543149 PMC5885018

[64] Zhou Z Alikhan NF Sergeant MJ Luhmann N Vaz C Francisco AP. GrapeTree: visualization of core genomic relationships among 100,000 bacterial pathogens. Genome Res. (2018) 28:1395–404. 10.1101/gr.232397.117 30049790 PMC6120633

[65] Uelze L Borowiak M Deneke C Szabo I Fischer J Tausch SH. Performance and accuracy of four open-source tools for in silico serotyping of salmonella spp. based on whole-genome short-read sequencing data. Appl Environ Microbiol. (2020) 86:19. 10.1128/AEM.02265-19 31862714 PMC7028957

[66] Banerji S Simon S Tille A Fruth A Flieger A. Genome-based Salmonella serotyping as the new gold standard. Sci Rep. (2020) 10:4333. 10.1038/s41598-020-61254-1 32152449 PMC7062728

[67] European Nucleotide Archive. Available online at: https://www.ebi.ac.uk/genomes/bacteria.html

[68] Kauffman G. Kauffmann white scheme. J Acta Path Microbiol Sci. (1974) 61:385.

[69] Duong VT The HC Nhu TDH Tuyen HT Campbell JI Minh PV. Genomic Serotyping, Clinical Manifestations, and Antimicrobial Resistance of Nontyphoidal Salmonella Gastroenteritis in Hospitalized Children in Ho Chi Minh City, Vietnam. J Clin Microbiol. (2020) 58:1645. 10.1128/JCM.01465-20 32907994 PMC7685882

[70] Parisi A Phuong TLT Mather AE Jombart T Tuyen HT Lan NPH. The role of animals as a source of antimicrobial resistant nontyphoidal Salmonella causing invasive and non-invasive human disease in Vietnam. Infect Genet Evol. (2020) 85:104534. 10.1016/j.meegid.2020.104534 32920195 PMC7705210

[71] Chattaway MA Langridge GC Wain J. Salmonella nomenclature in the genomic era: a time for change. Sci Rep. (2021) 11:7494. 10.1038/s41598-021-86243-w 33820940 PMC8021552

[72] Doyle RM O'Sullivan DM Aller SD Bruchmann S Clark T Coello Pelegrin A. Discordant bioinformatic predictions of antimicrobial resistance from whole-genome sequencing data of bacterial isolates: an inter-laboratory study. Microb Genom. (2020) 6:335. 10.1099/mgen.0.000335 32048983 PMC7067211

[73] Wu B Ed-Dra A Pan H Dong C Jia C Yue M. Genomic investigation of salmonella isolates recovered from a pig slaughtering process in Hangzhou, China. Front Microbiol. (2021) 12:704636. 10.3389/fmicb.2021.704636 34305874 PMC8298193

[74] Liu Q Chen W Elbediwi M Pan H Wang L Zhou C. Characterization of Salmonella Resistome and Plasmidome in Pork Production System in Jiangsu, China. Front Vet Sci. (2020) 7:617. 10.3389/fvets.2020.00617 33062654 PMC7517575

[75] Zhou K Tao Y Han L Ni Y Sun J. Piperacillin-Tazobactam (TZP) Resistance in escherichia coli due to hyperproduction of tem-1 beta-lactamase mediated by the promoter Pa/Pb. Front Microbiol. (2019) 10:833. 10.3389/fmicb.2019.00833 31040841 PMC6476967

[76] Deekshit VK Kumar BK Rai P Srikumar S Karunasagar I Karunasagar I. Detection of class 1 integrons in Salmonella Weltevreden and silent antibiotic resistance genes in some seafood-associated nontyphoidal isolates of Salmonella in south-west coast of India. J Appl Microbiol. (2012) 112:1113–22. 10.1111/j.1365-2672.2012.05290.x 22443444

[77] Enne VI Delsol AA Roe JM Bennett PM. Evidence of antibiotic resistance gene silencing in Escherichia coli. Antimicrob Agents Chemother. (2006) 50:3003–10. 10.1128/AAC.00137-06 16940095 PMC1563515

[78] Elbediwi M Beibei W Pan H Jiang Z Biswas S Li Y. Genomic characterization of mcr-1-carrying Salmonella enterica Serovar 4,[5],12:i:- ST 34 clone isolated from pigs in China. Front Bioeng Biotechnol. (2020) 8:663. 10.3389/fbioe.2020.00663 32714906 PMC7344297

[79] Li XP Fang LX Song JQ Xia J Huo W Fang JT. Clonal spread of mcr-1 in PMQR-carrying ST34 Salmonella isolates from animals in China. Sci Rep. (2016) 6:38511. 10.1038/srep38511 27917926 PMC5137007

[80] Arnott A Wang Q Bachmann N Sadsad R Biswas C Sotomayor C. Multidrug-resistant salmonella enterica 4, [5], 12:i:- Sequence Type 34, New South Wales, Australia, 2016–2017. Emerg Infect Dis. (2018) 24:751–3. 10.3201/eid2404.171619 29553318 PMC5875280

[81] Elnekave E Hong S Mather AE Boxrud D Taylor AJ Lappi V. Salmonella enterica Serotype 4, [5], 12:i:- in Swine in the United States Midwest: an emerging multidrug-resistant clade. Clin Infect Dis. (2018) 66:877–85. 10.1093/cid/cix909 29069323

[82] Litrup E Kiil K Hammerum AM Roer L Nielsen EM Torpdahl M. Plasmid-borne colistin resistance gene mcr-3 in Salmonella isolates from human infections, Denmark, 2009–17. Euro Surveill. (2017) 22:30587. 10.2807/1560-7917.ES.2017.22.31.30587 28797325 PMC5553060

[83] Sun RY Ke BX Fang LX Guo WY Li XP Yu Y. Global clonal spread of mcr-3-carrying MDR ST34 Salmonella enterica serotype Typhimurium and monophasic 1,4,[5],12:i:- variants from clinical isolates. J Antimicrob Chemother. (2020) 75:1756–65. 10.1093/jac/dkaa115 32274508

[84] Mulvey M Bharat A Boyd D Irwin R Wylie J. Characterization of a colistin-resistant Salmonella enterica 4, [5], 12:i:- harbouring mcr-3.2 on a variant IncHI-2 plasmid identified in Canada. J Medi Microbiol. (2018) 67:854. 10.1099/jmm.0.000854 30351266

[85] Biswas S Li Y Elbediwi M Yue M. Emergence and Dissemination of mcr-Carrying Clinically Relevant Salmonella Typhimurium Monophasic Clone ST34. Microorganisms. (2019) 7:298. 10.3390/microorganisms7090298 31466338 PMC6780495

[86] Nhung NT Van NTB Cuong NV Duong TTQ Nhat TT Hang TTT. Antimicrobial residues and resistance against critically important antimicrobials in non-typhoidal Salmonella from meat sold at wet markets and supermarkets in Vietnam. Int J Food Microbiol. (2018) 266:301–9. 10.1016/j.ijfoodmicro.2017.12.015 29275223 PMC5783717

[87] Gonzalez-Santamarina B Garcia-Soto S Dang-Xuan S Abdel-Glil MY Meemken D Fries R. Genomic characterization of multidrug-resistant salmonella serovars derby and rissen from the pig value chain in Vietnam. Front Vet Sci. (2021) 8:705044. 10.3389/fvets.2021.705044 34513973 PMC8429848

[88] Gonzalez-Santamarina B Busch A Garcia-Soto S Abdel-Glil MY Linde J Fries R. Draft genome sequence of multi-resistant Salmonella enterica subsp. enterica serovar Rissen strain 19CS0416 isolated from Vietnam reveals mcr-1 plasmid mediated resistance to colistin already in 2013. J Genomics. (2020) 8:76–9. 10.7150/jgen.42790 32817764 PMC7425045

[89] Critically Important Antimicrobials For Human Medicine 6th revision. Licence: CC BY-NC-SA 3.0 IGO. Geneva: World Health Organisation (2019).

[90] European Food Safety A European Centre for Disease P Control. The European Union summary report on antimicrobial resistance in zoonotic and indicator bacteria from humans, animals, and food in 2015. EFSA J. (2017) 15:e04694. 10.2903/j.efsa.2017.4694 32625402 PMC7009883

[91] Nguyen KV Thi Do NT Chandna A Nguyen TV Pham CV Doan PM. Antibiotic use and resistance in emerging economies: a situation analysis for Viet Nam. BMC Public Health. (2013) 13:1158. 10.1186/1471-2458-13-1158 24325208 PMC4116647

[92] Lettini AA Vo Than T Marafin E Longo A Antonello K Zavagnin P. Distribution of Salmonella Serovars and antimicrobial susceptibility from poultry and swine farms in Central Vietnam. Zoonoses Public Health. (2016) 63:569–76. 10.1111/zph.12265 26952244

[93] European Food Safety A European Centre for Disease P Control. The European union summary report on antimicrobial resistance in zoonotic and indicator bacteria from humans, animals and food in 2017/2018. EFSA J. (2020) 18:e06007. 10.2903/j.efsa.2020.6007 32874244 PMC7448042

