# Supplementary figures and images for: Analysis of Antimicrobial Resistance in Non-typhoidal Salmonella Collected From Pork Retail Outlets and Slaughterhouses in Vietnam Using Whole Genome Sequencing
Source: Front Vet Sci. 2022 Mar 29;9:816279. doi: 10.3389/fvets.2022.816279 (PMC9002014; doi:10.3389/fvets.2022.816279)

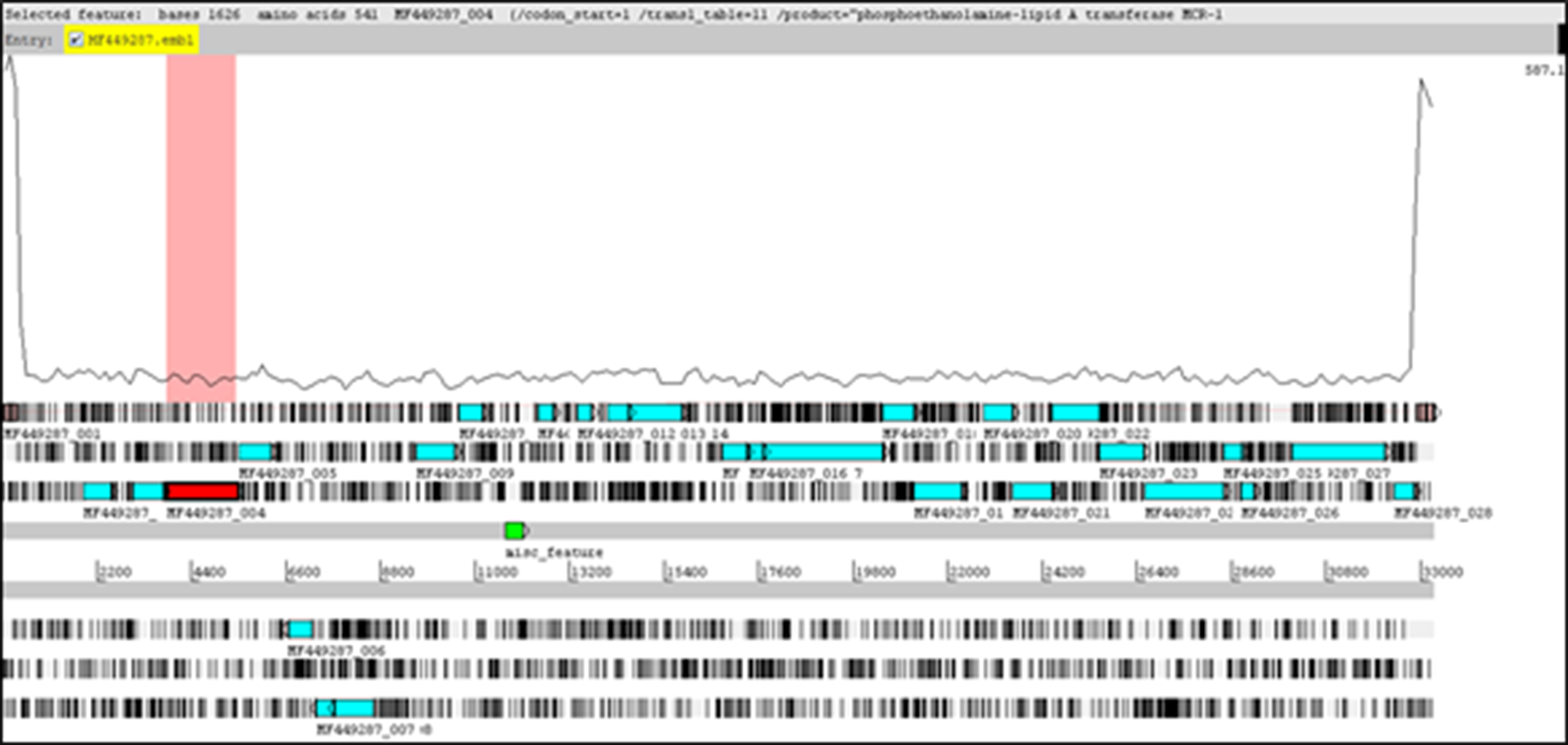

Supplement: Supplementary file 1 [file Image_1.TIF]

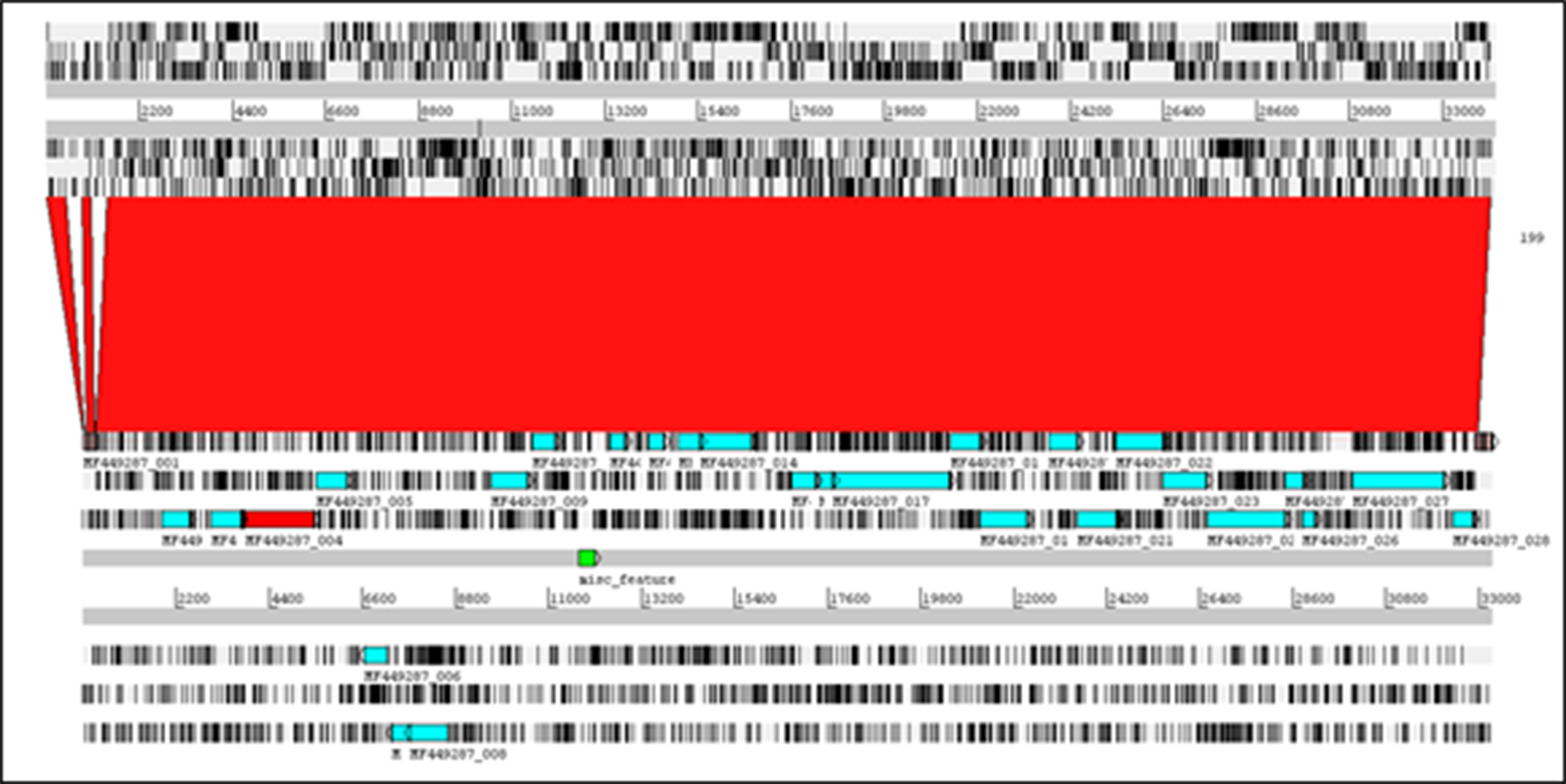

Supplement: Supplementary file 2 [file Image_2.TIF]

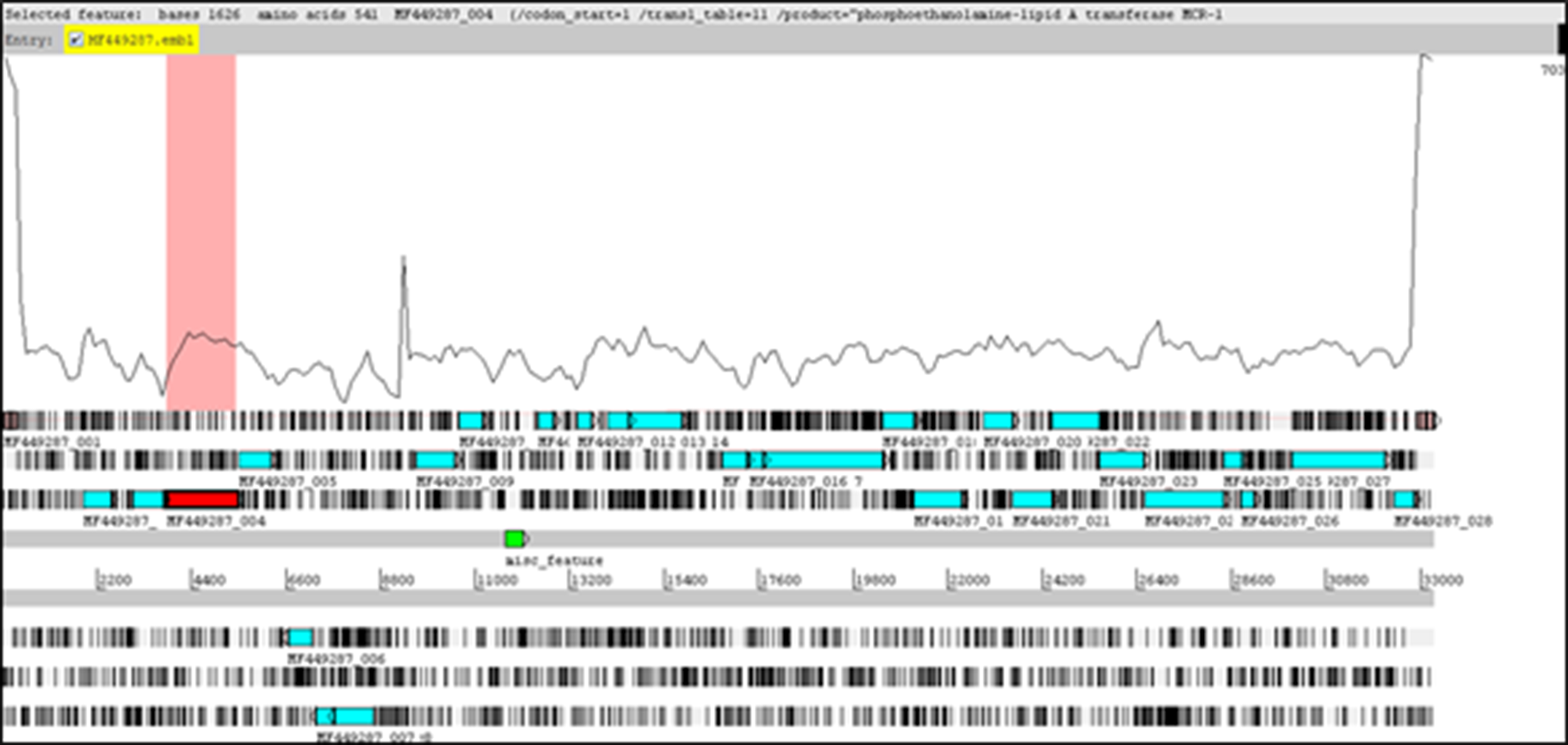

Supplement: Supplementary file 3 [file Image_3.TIF]

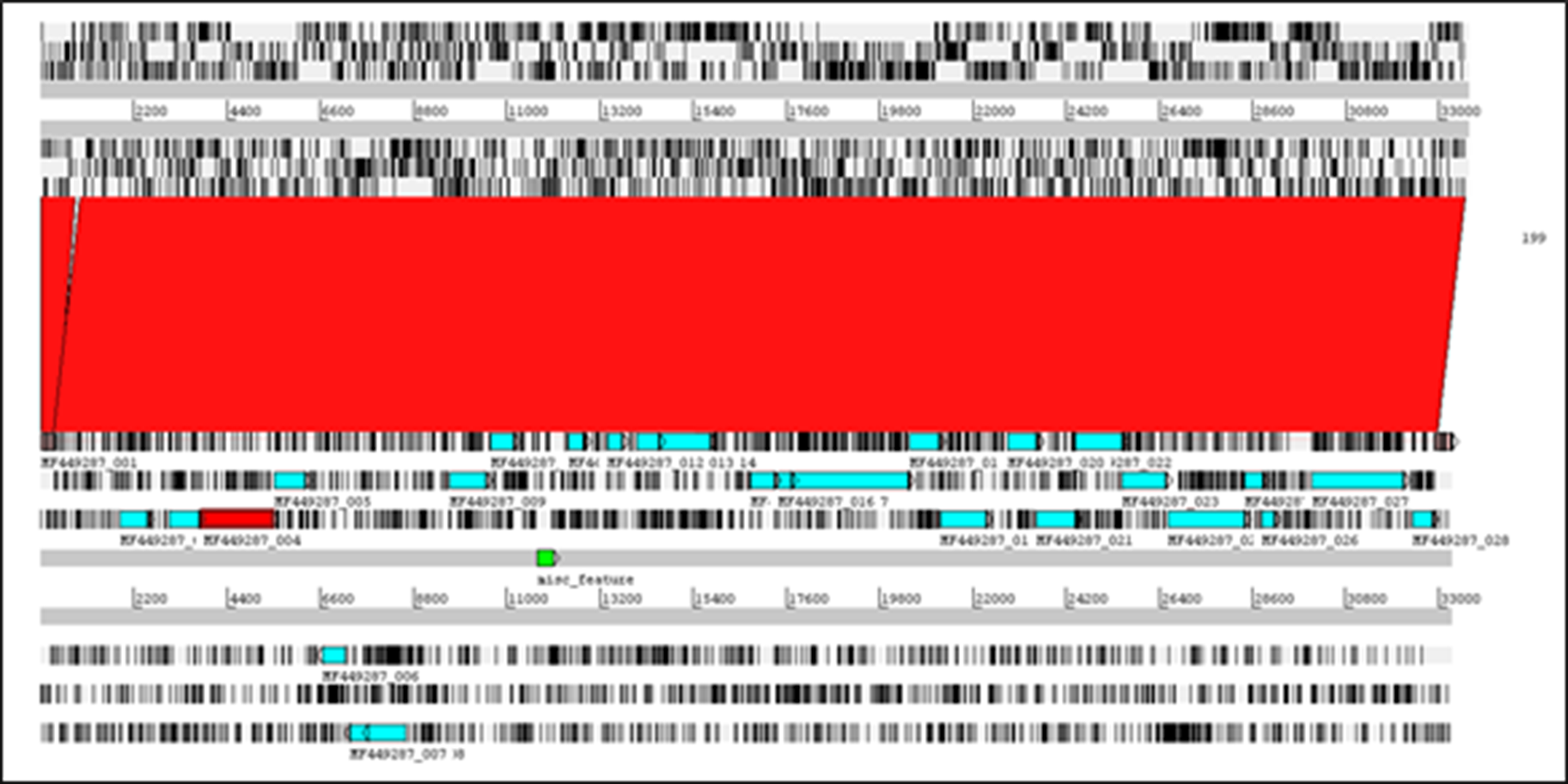

Supplement: Supplementary Figure 1 — Comparison between mcr-1 positive non-typhoid Salmonella with reference mcr-1 plasmid plMBC (MF449287.2). CDS MF449287_004 (red) indicates location of mcr-1. (A) SP90 fastq reads mapped to pIMBC plasmid sequence; (B) ACT comparison of SP90 draft genome with pIMBC plasmid sequence; (C) SP164 fastq reads mapped to pIMBC plasmid sequence; (D) ACT comparison of SP164 draft genome with pIMBC plasmid sequence. [file Image_4.TIF]
